# Coexisting Phases in NaNbO_3_ Thin Films Influenced by Epitaxial Strain and Size Effects

**DOI:** 10.1002/advs.202510099

**Published:** 2025-10-14

**Authors:** Aarushi Khandelwal, Kevin J. Crust, Reza Ghanbari, Yijun Yu, Ruijuan Xu, Harold Y. Hwang

**Affiliations:** ^1^ Stanford Institute for Materials and Energy Sciences SLAC National Accelerator Laboratory Menlo Park CA 94025 USA; ^2^ Department of Applied Physics Stanford University Stanford CA 94305 USA; ^3^ Department of Physics Stanford University Stanford CA 94305 USA; ^4^ Department of Materials Science and Engineering North Carolina State University Raleigh NC 27695 USA

**Keywords:** antiferroelectrics, dielectric capacitors, phase coexistence, size effects, sodium niobate

## Abstract

Antiferroelectrics are a promising class of materials for applications in capacitive energy storage and multi‐state memory, but comprehensive control of their functional properties requires further research. In thin films, epitaxial strain and size effects are important tuning knobs but difficult to probe simultaneously due to low critical thicknesses of common lead‐based antiferroelectrics. Antiferroelectric NaNbO_3_ enables opportunities for studying size effects under strain, but electrical properties of ultra‐thin films have not been thoroughly investigated due to materials challenges. Here, high‐quality, epitaxial, coherently‐strained NaNbO_3_ films are synthesized from 35‐ to 250‐ nm thickness, revealing a transition from a fully ferroelectric state to coexisting ferroelectric and antiferroelectric phases with increasing thickness. The electrical performance of this phase coexistence is analyzed through positive‐up negative‐down and first‐order reversal curve measurements. Further increasing thickness leads to a fully ferroelectric state due to a strain relief mechanism that suppresses the antiferroelectricity. The potential of engineering competing ferroic orders in NaNbO_3_ for multiple applications is evaluated, reporting significantly enhanced recoverable energy density (20.6 J cm^−3^ at 35 nm) and energy efficiency (90% at 150 nm) relative to pure bulk NaNbO_3_ as well as strong retention and fatigue performance with multiple accessible polarization states in the intermediate thickness films.

## Graphical abstract

Coherently strained NaNbO_3_ thin films are synthesized across a range of thicknesses, enabling the study of size effects under epitaxial strain in antiferroelectric thin films without relaxation. Competing antiferroelectric‐ferroelectric phases are observed, leading to the stabilization of phase coexistence, multistate switching with strong retention and fatigue performance, and potential avenues to boost energy storage properties.

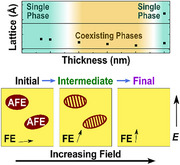

## 1 Introduction

Antiferroelectric (AFE) materials, which possess antiparallel electric dipoles that can be reoriented into a parallel ferroelectric (FE) state under a sufficiently strong electric field, are a promising class of materials for applications like capacitive energy storage, multistate random access memory, actuation, and electrocaloric cooling due to the large structural and property changes that occur during the dipole reorientation process.^[^ ^1^, ^2^, ^3^ ^]^ Although a theoretical framework for antiferroelectricity was established in 1951,^[^ ^4^ ^]^ a complete understanding of its underlying mechanisms and control of its functional properties remains lacking. This is particularly true in thin films, which offer significant engineering opportunities and can manifest drastically different behaviors from bulk materials.^[^ ^1^, ^3^ ^]^ Most AFE thin film studies thus far are of lead‐based materials like PbZrO_3_, which have a significant lattice mismatch from commercially available substrates such as SrTiO_3_ or DyScO_3_. Epitaxial strain applied by substrates is an effective knob for tuning the properties of AFE thin films, but the large mismatch leads to relatively low critical thicknesses, typically a few tens of nm, above which the strain is relaxed.^[^ ^5^, ^6^, ^7^ ^]^ This both limits the practical applications for which strain engineering can be utilized, as properties like leakage current and coercive field typically perform worse in extremely thin films, and also prevents the study of size effects, another valuable thin film tuning knob, under strain.

NaNbO_3_ is a promising alternative to the predominant lead‐based AFEs, displaying a complex phase diagram with multiple competing ferroic orders stabilized by tuning temperature and applied electric field.^[^ ^8^, ^9^, ^10^, ^11^ ^]^ Bulk NaNbO_3_ is an AFE material with orthorhombic *Pbcm* space group at room temperature that undergoes an irreversible phase transition to a polar FE phase with orthorhombic *Pmc2_1_* space group under an applied electric field, due to the small energy difference between these phases.^[^ ^10^ ^]^ Its lattice parameters are also better matched with commercial substrates, allowing thin films to remain coherently strained up to several hundreds of nm in thickness on SrTiO_3_.^[^ ^12^ ^]^

Despite these opportunities, previous studies on strain engineering or size effects in NaNbO_3_ thin films have been mostly limited to theoretical or structural analyses, with significant questions remaining about the nature of their field‐induced transitions. Various FE ground states have been predicted through strain‐induced phase transitions, including a monoclinic *Cc* phase with simultaneous in‐plane and out‐of‐plane polarizations for small magnitudes of strain, which is distinct from both the low temperature (*F3c*) or room temperature field‐induced (*Pmc2_1_*) bulk FE phases.^[^ ^10^, ^13^, ^14^, ^15^, ^16^ ^]^ Strain has been experimentally verified to modify the room temperature ground state of NaNbO_3_, with a FE ground state observed in films with large magnitudes of tensile strain, using Raman spectroscopy and piezoresponse force microscopy (PFM), as well as an AFE ground state identified in films with large compressive strain using a superlattice reflection from unit cell quadrupling in the AFE phase.^[^ ^12^, ^17^, ^18^ ^]^ On SrTiO_3_, which applies small degree of strain that can either be compressive or tensile depending on the film orientation, both FE and AFE room temperature ground states have been reported.^[^ ^19^, ^20^, ^21^ ^]^ Size effects have also been shown to alter the phase balance in the absence of epitaxial strain, with freestanding NaNbO_3_ membranes transitioning from a single FE phase to coexisting FE and AFE phases with increasing thickness.^[^ ^22^ ^]^

Understanding the electrical properties and field‐induced transitions in NaNbO_3_ has been mostly limited to bulk samples due to high leakage currents in thin films. Such leakage has been attributed to point defects in the system, due to sodium vacancies from the highly volatile alkali component or oxygen vacancies akin to other oxide perovskite thin films.^[^ ^23^ ^]^ This has limited measurements of well‐defined polarization – electric field (*P‐E)* hysteresis loops for thin films below 200 nm and made it difficult to distinguish between FE and AFE characteristics in electrical characterization of thinner films.^[^ ^19^, ^24^, ^25^ ^]^ Even for thin films with structurally identified AFE ground states, it is unclear whether they will undergo the same irreversible phase transition as bulk or maintain their AFE character under applied fields, and any influence of size effects on these transitions is currently unknown.

Electrical characterization of NaNbO_3_ thin films is also essential for determining the potential of NaNbO_3_ for applications like capacitive energy storage or multistate switching. The main parameters in capacitive energy storage, recoverable energy density and energy efficiency, are directly extracted from *P‐E* hysteresis loops by various integrations of the curves and thus optimizing materials toward specific loop shapes is an area of active research.^[^ ^26^ ^]^ Most work in this direction on bulk NaNbO_3_‐based systems has focused on utilizing chemical substitution to destabilize the FE phase relative to the AFE phase by altering the tolerance factor and polarizability of the B‐site cations, but limitations on breakdown fields and energy storage densities persist.^[^ ^27^, ^28^, ^29^, ^30^ ^]^

On the other hand, multistate switching capabilities, desirable for high density memory and neuromorphic computing applications, are frequently associated with some degree of pinching in *P‐E* hysteresis loops and evaluated with pulsed electrical measurements such as positive‐up negative‐down (PUND) tests to identify stable states with good retention and fatigue tolerance. Several paths have been explored to create these multi‐state systems from other bistable ferroelectrics, including controlling switching pathways,^[^ ^31^, ^32^ ^]^ defect engineering to alter the nucleation processes,^[^ ^33^ ^]^ or utilizing coexisting structural variants,^[^ ^34^, ^35^, ^36^ ^]^ with NaNbO_3_ being a promising candidate for the last pathway given its numerous competing ferroic orders.

In this work, we investigate the impact of size effects under epitaxial strain on NaNbO_3_ thin films from 35 to 250 nm on SrTiO_3_ (001) substrates and achieve well‐defined *P‐E* loops across the entire thickness range via precise control over leakage currents through detailed growth optimization. We observe the coexistence of FE and AFE orders at intermediate thicknesses through detailed electrical and X‐ray based structural characterization techniques and reveal a stable intermediate state during electrical switching at these thicknesses. A switching mechanism of this state is proposed and verified through first‐order reversal curve (FORC) measurements, including a reversible AFE‐FE transition that is not typically observed in bulk NaNbO_3_. The balance between the coexisting phases is shown to be altered by a mixed‐orientation strain relief mechanism which suppresses antiferroelectricity in the thickest film. Finally, we evaluate the application potential of miniaturizing the epitaxially strained NaNbO_3_ thin films, with decreasing thickness boosting the recoverable energy density in the thinnest film to 20.6 J cm^−1^ and operation in the intermediate state at intermediate thicknesses yielding an energy storage efficiency of 90%, both records for pure NaNbO_3_. The intermediate state at intermediate thicknesses is also shown to host multiple polarization states with good retention and nearly fatigue‐free performance for potential non‐volatile memory applications.

## 2 Results and Discussion

### 2.1 Structural Characterization of NaNbO_3_ Capacitor Heterostructures

To investigate the impact of size effects under epitaxial strain on NaNbO_3_ films, we synthesized La_0.7_Sr_0.3_MnO_3_ / NaNbO_3_ / La_0.7_Sr_0.3_MnO_3_ tri‐layer heterostructures on single‐crystalline SrTiO_3_ (001) substrates by pulsed laser deposition, with the NaNbO_3_ thickness ranging from 35 to 250 nm. The top La_0.7_Sr_0.3_MnO_3_ layer was etched after photolithographic patterning to create symmetric parallel‐plate capacitor structures with 100 µm diameter circular top electrodes (Experimental Section). To mitigate the high leakage currents typically prevalent in NaNbO_3_, we optimized the growth conditions to minimize leakage while maintaining good crystalline quality of the NaNbO_3_ films (Figure S1, Supporting Information).^[^ ^19^, ^25^, ^37^ ^]^

**Figure 1:**
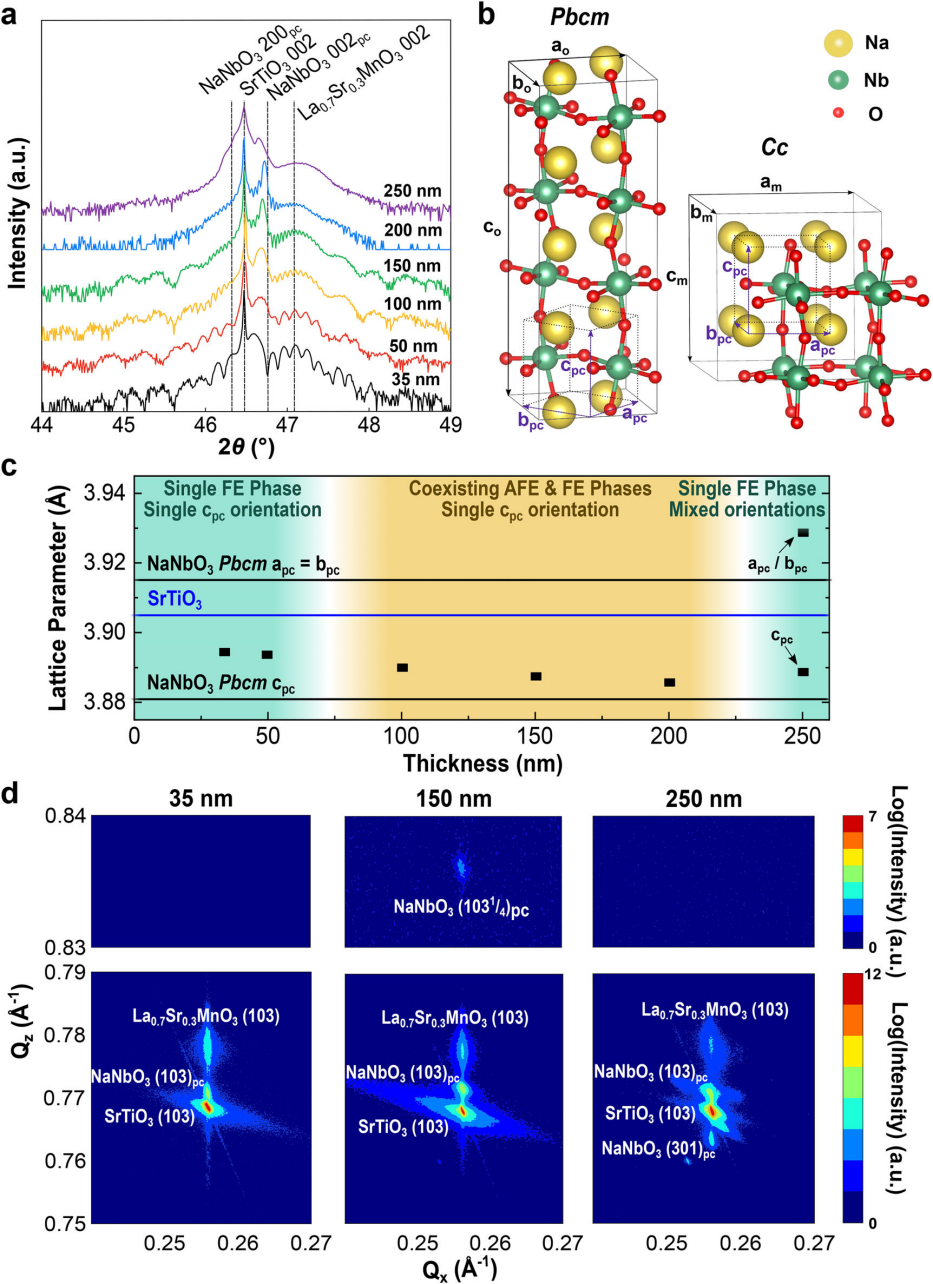
Structural characterization of La_0.7_Sr_0.3_MnO_3_ / NaNbO_3_ / La_0.7_Sr_0.3_MnO_3_ heterostructures on SrTiO_3_ (001) substrates. a) X‐ray *θ*–2*θ* line scans of La_0.7_Sr_0.3_MnO_3_ / NaNbO_3_ / La_0.7_Sr_0.3_MnO_3_ heterostructures on SrTiO_3_ (001) substrates with varying NaNbO_3_ thicknesses. b) Schematic of the antiferroelectric *Pbcm* structure, displaying a quadrupling of the unit cell leading to a quarter‐order diffraction peak, and the ferroelectric *Cc* structure, which only undergoes a doubling of the unit cell. c) Evolution of out‐of‐plane NaNbO_3_ lattice parameters with varying film thickness. The solid lines represent the bulk lattice parameters of the room temperature *Pbcm* structure of NaNbO_3_ as well as SrTiO_3_. The background is colored based on the thicknesses exhibiting a quarter‐order diffraction peak in Figure S2 (Supporting Information). d) X‐ray reciprocal space maps about the (103) diffraction condition for heterostructures with 35, 150, and 250 nm NaNbO_3_.

High crystalline quality was confirmed for all samples, evidenced by the 002 *θ–*2*θ* X‐ray diffraction (XRD) patterns (**Figure** **1**a) that exhibit prominent Laue oscillations in all but the thickest film. The lack of Laue oscillations in the 250 nm film can be attributed to either the resolution limits of our diffractometer or a possible small increase in disorder in the thickest sample, which could also affect the crystallinity of the top La_0.7_Sr_0.3_MnO_3_ layer. All films display a NaNbO_3_ film peak at a higher 2*θ* angle than SrTiO_3_, corresponding to a lattice parameter between 3.886 and 3.894 Å, with the thickest film also exhibiting an additional lower angle peak with a lattice parameter of 3.929 Å. Comparing the reduced pseudocubic cell of bulk NaNbO_3_ (schematic in Figure 1b,^[^ ^13^, ^22^ ^]^ *a_pc_* = *b_pc_* = 3.915 Å, *c_pc_* = 3.881 Å) and the extracted lattice parameters (Figure 1c), we note that the orientation of the NaNbO_3_ film appears to change with increasing thickness from *c_pc_*‐ oriented to a mixture of structural variants with *c_pc_*‐ and *a_pc_* / *b_pc_*‐ orientations.^[^ ^38^ ^]^ Although bulk lattice parameters of the ferroelectric *Cc* phase are not available, the predicted values are a slight expansion of the *Pbcm* lattice, consistent with our results.^[^ ^13^ ^]^ Thus, regardless of the FE or AFE phase, the *c_pc_‐* orientation, with bulk in‐plane lattice parameters of ≈3.915 Å, experiences biaxial compressive in‐plane strain on SrTiO_3_ substrates. In contrast, the *a_pc_* / *b_pc_‐* orientation, with in‐plane lattice parameters of ≈3.915 and ≈3.881 Å, undergoes anisotropic strain – compressive along one in‐plane direction and tensile along the other – resulting in a lower compressive strain state. This structural evolution serves as a strain relief mechanism, lowering the increasing strain energy of thicker films through the combined compressive and tensile strains of the mixed *c_pc_*‐ and *a_pc_* / *b_pc_*‐ orientations.^[^ ^12^ ^]^ We also note that the recently reported *Pc* or *Cm* structures could provide an alternative mechanism for this secondary peak in the thickest sample.^[^ ^16^, ^39^ ^]^

We conducted X‐ray reciprocal space mapping (RSM) about the SrTiO_3_ (103) diffraction condition to further understand the NaNbO_3_ structure and confirmed that all films are coherently epitaxially strained (Figure 1d; Figure S2, Supporting Information). Comparing the various thicknesses, an additional NaNbO_3_ (301)_pc_ peak can be seen only in the 250 nm sample, providing further evidence of the transition to a mixed orientation structure. We also observed the emergence of a quarter‐order peak in the intermediate thickness range from 100 to 200 nm. Such a quarter‐order diffraction peak is a characteristic feature of the AFE phase in NaNbO_3_, resulting from the fourfold multiplicity of the *Pbcm* unit cell perpendicular to the polarization direction, whereas the *Cc* structure only undergoes a unit cell doubling (Figure 1b). These results indicate that thinner films only possess FE order, with AFE order appearing as thickness increases, aligned with previous observations in NaNbO_3_ freestanding membranes.^[^ ^22^ ^]^ Notably, a splitting of the main NaNbO_3_ film peak from this phase coexistence could not be observed in either the XRD line scans or RSMs, likely indicating that the phases have similar lattice parameters. As thickness further increases, the AFE phase disappears completely in the 250 nm film. We attribute this to the onset of tensile strain discussed above, which is known to stabilize the FE phase in NaNbO_3_ and is experienced by the shorter in‐plane axis of the *a_pc_* / *b_pc_‐* orientation in the thickest film.^[^ ^12^, ^17^, ^18^ ^]^

### 2.2 Electrical Characterization Revealing a Stable Intermediate State During Switching

**Figure 2:**
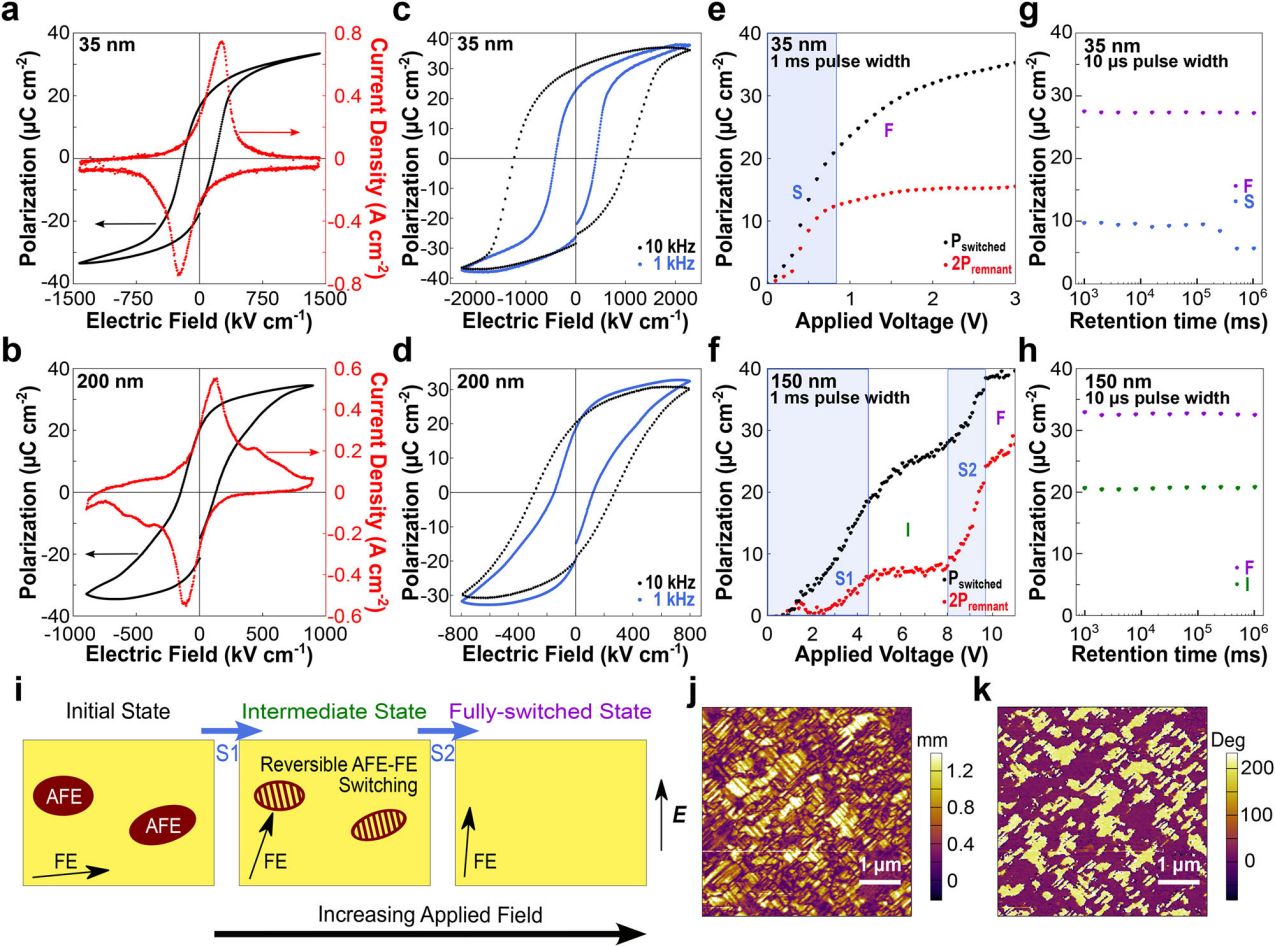
Evidence of a stable intermediate state at intermediate thicknesses. a,b) Ferroelectric hysteresis loop and corresponding switching current curve measured at 1 kHz for (a) 35 and (b) 200 nm NaNbO_3_. c,d) Frequency dependence of ferroelectric hysteresis loops for (c) 35 and (d) 200 nm NaNbO_3_. e,f) Evolution of switched and remnant polarizations from PUND measurements as a function of applied voltage with a 1 ms pulse width for (e) 35 and (f) 150 nm NaNbO_3_. *P_switched_* is measured directly from the pulse train in Figure S8 (Supporting Information), while *2P_remnant_* is calculated as *P_switched_* – *P_unswitched_*. g,h) Retention measurements probing the polarization states in different voltage regimes with 10 µs pulse widths for (g) 35 nm [1 V for sub‐switching (*S*); 5 V for fully switched (*F*)] and (h) 150 nm NaNbO_3_ [8 V for intermediate state (*I*); 15 V for fully switched (*F*)]. i) Schematic of possible switching mechanism under increasing applied field in intermediate thickness films with mixed AFE and FE phases. Labels *S1*, Intermediate (*I*), *S2*, and Fully‐switched (*F*) correspond to the voltage regimes in (f). j,k) Lateral PFM (j) amplitude and (k) phase images of the 100 nm NaNbO_3_ film exhibiting a strong in‐plane FE response without phase segregation.

We further study how the thickness‐dependent structural evolution and competing AFE and FE phases influence the electrical properties of NaNbO_3_ films. Although leakage currents typically increase dramatically with reduced thickness,^[^ ^40^ ^]^ we maintain low leakage currents across the entire thickness range (Figure S3, Supporting Information), allowing us to probe well‐defined *P‐E* loops in all samples. Both the 35 and 250 nm films exhibit standard ferroelectric hysteresis loops and similar saturation polarizations (**Figure** **2**a,c; Figures S4 and S5, Supporting Information), with the thinner film displaying significantly reduced remnant polarization and more slanted hysteresis loops. This variation between the 35 and 250 nm samples can be attributed to the changes in film orientation with thickness and is directly observable through piezoresponse force microscopy (Figure S6, Supporting Information). For the 35 nm samples, the polarization is strongest along the in‐plane [110]_pc_ direction with a smaller out‐of‐plane polarization, providing further evidence for the monoclinic *Cc* phase. In contrast, the 250 nm sample has two laterally segregated regions: a region at lower height with the same predominately in‐plane polarization and a region at a higher height with relatively stronger out‐of‐plane polarization. The difference in height between these regions matches the expected value given the thickness of the film and the difference in out‐of‐plane lattice parameter between the orientations from Figure 1c.

For the intermediate thickness samples (Figure 2b; Figure S4, Supporting Information), we measure pinched hysteresis loops accompanied by multiple peaks in the corresponding switching current curve, which is most evident in the 200 nm sample shown in Figure 2 but still observable at other intermediate thicknesses included in the Supporting Information. The degree of pinching is found to be more pronounced as the frequency decreases (Figure 2d; Figure S5, Supporting Information), ruling out defect‐induced pinching in these films and instead suggesting that the resultant pinched hysteresis loops are a combination of FE loops and AFE double hysteresis loops, similar to previous work in mixed‐phase NaNbO_3_ systems.^[^ ^24^, ^29^, ^41^ ^]^ Additionally, while the extracted relative permittivity values ranging between 150 and 200 for the 35 and 250 nm films are similar to previously reported values for NaNbO_3_,^[^ ^25^, ^42^, ^43^ ^]^ the intermediate thickness films show a significant increase up to ≈670 (Figure S7, Supporting Information), as expected due to the phase coexistence with multiple ferroic orders.^[^ ^44^ ^]^

To better understand the thickness dependence of the electrical behavior of films, we conducted PUND and retention measurements, with the corresponding pulse sequences in Figure S8 (Supporting Information). We carried out PUND measurements with 1 ms pulse width as a function of pulse voltage and measured switched and unswitched polarizations, from which we extract the remnant polarization: 2*P_remnant_* = *P_switched_* – *P_unswitched_*. PUND measurements on the 35 nm film (Figure 2e) reveal two regimes in the voltage dependence: a sub‐switching regime (*S*) at lower voltages, where polarization increases with voltage, and a fully‐switched state (*F*) at higher voltages, where the remnant polarization plateaus. The slower, continued increase of switched polarization in the fully‐switched state is attributed to leakage currents. Corresponding retention measurements at 1 and 5 V with a pulse width of 10 µs (Figure 2g) show that the polarization in the sub‐switching regime exhibits poorer retention than in the fully‐switched state. In contrast, PUND measurements on the intermediate thickness of 150 nm (Figure 2f) exhibit multistate switching with an additional, stable intermediate state, resulting in four distinct regimes in the voltage dependence: two sub‐switching regimes (*S1* and *S2*) as well as intermediate (*I*) and fully‐switched (*F*) states. Corresponding retention measurements (Figure 2h) taken at 8 and 15 V show that the polarization of both the intermediate and fully‐switched states are equivalently stable.

What causes this stable intermediate state in intermediate thickness films? Based on the presence of the AFE phase in intermediate thickness films from structural characterization, here we propose a potential mechanism for the multistate switching arising from competition between the FE and AFE ferroic orders. Figure 2i illustrates a possible schematic for the film response under increasing out‐of‐plane electric field. The proposed initial state at zero field has randomly distributed clusters of AFE phase within the FE phase, which has a canted, predominately in‐plane polarization, based on both the structural characterization and the PFM measurements (Figure 2j,k; Figure S9, Supporting Information) which do not show lateral segregation between AFE and FE phases. In particular, the *Cc* phase is predicted to have a stronger in‐plane polarization along [110]_pc_ with a weaker out‐of‐plane polarization when strained to SrTiO_3_,^[^ ^13^ ^]^ matching our experimental results.

Upon initial field application, the FE phase response dominates, leading to increasing switched and remnant polarizations throughout the sub‐switching regime *S1* (Figure 2f). At a critical field, dependent on the film thickness but independent of pulse width (Figures S10 and S11, Supporting Information), the film transitions to the intermediate state *I* where the applied electric field triggers a reversible AFE‐to‐FE transition that does not increase the remnant polarization, since removing the applied field causes an immediate relaxation back to the coexistence of AFE and FE phases, leading to the plateau in remnant polarization in Figure 2f. At higher applied fields, the FE‐to‐AFE transition becomes less stable in sub‐switching regime *S2*, leading to increases in both the switched and remnant polarizations until saturation in the fully‐switched state (*F*). This change in the stability of the AFE‐FE transition was also observed in the field dependence of *P‐E* hysteresis (Figure S12, Supporting Information), where a small increase in the maximum applied field leads to a drastic increase in the remnant polarization for the intermediate thicknesses, indicating that the reverse FE‐to‐AFE transition has been destabilized. However, unlike bulk NaNbO_3_ ceramics,^[^ ^30^ ^]^ the AFE‐to‐FE transition is not irreversible after fields in the *S2* and *F* regime are applied: subsequent measurements with maximum fields in the intermediate regime still show reduced remnant polarization in both *P‐E* hysteresis and PUND measurements, indicating the films can be returned to a coexistence of AFE and FE phases. This can be directly observed in Figure S13 (Supporting Information), where PUND measurements were performed sequentially with the pulse voltage being varied from the intermediate state to the fully‐switched state and then back to the intermediate state. The ability to deterministically access either state verifies that reversibility of the AFE‐to‐FE transition has been maintained even after entering the fully‐switched state. Notably, a complete PUND pulse sequence (Figure S8, Supporting Information) was performed at each data point using a procedure adapted from a previous work,^[^ ^32^ ^]^ with the negative preset pulse providing a reversal field which is required to enable the FE‐to‐AFE transition from the fully‐switched state.

Thus, the reversibility of the AFE‐to‐FE transition is distinct between the intermediate state and the fully‐switched state. From the intermediate state, the transition is reversible upon removal of the applied field while from the fully‐switched state the transition becomes reversible only upon application of a reversal field, leading to the higher remnant polarization of the fully‐switched state. We note that the above discussion is somewhat phenomenological, and further work is needed to fully understand the microscopic mechanism of the intermediate state during switching.

**Figure 3:**
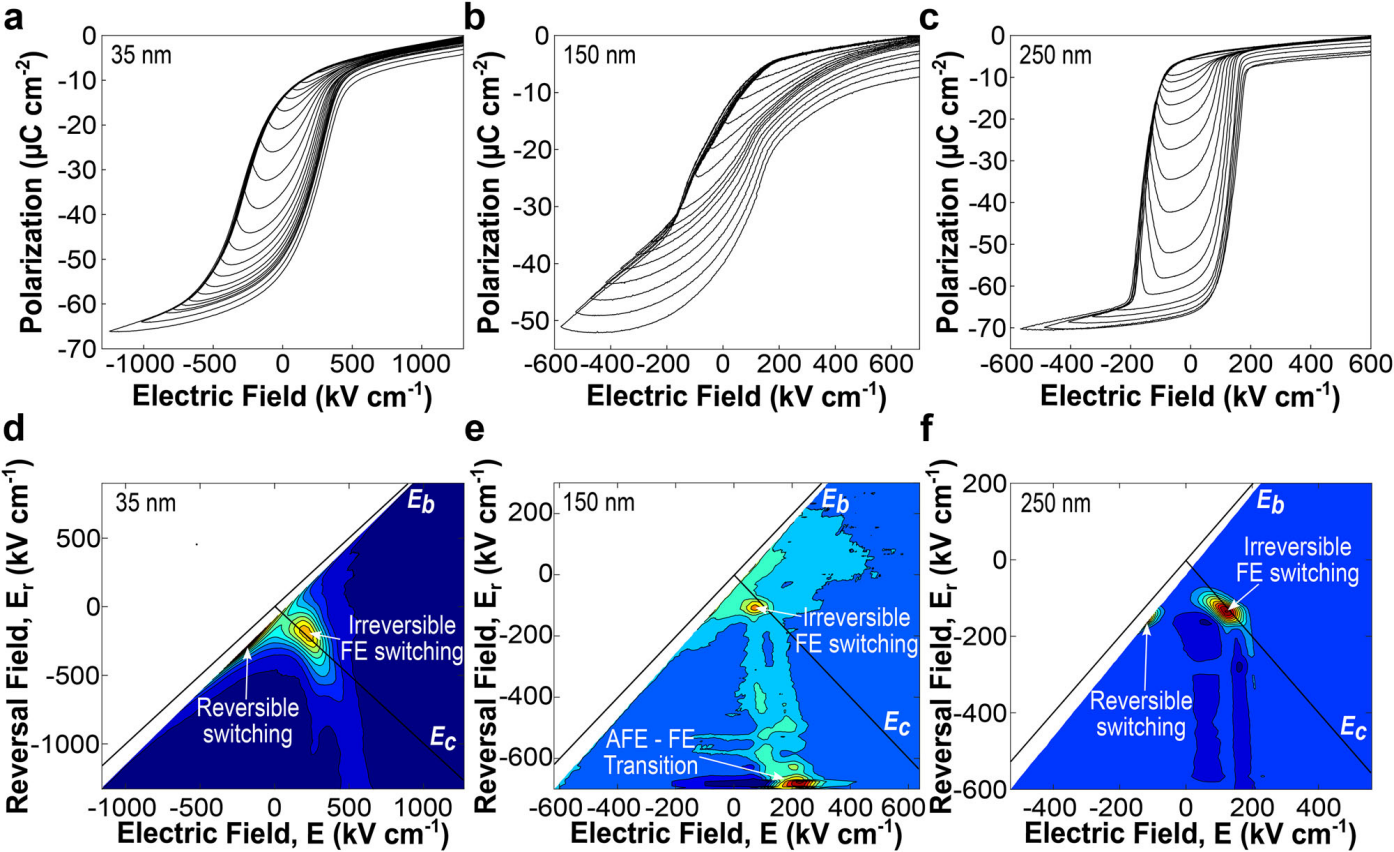
NaNbO_3_ switching characteristics from FORC measurements. a–c) Selected minor hysteresis loops measured from a positive saturating field to varying reversal fields *E_r_* for a) 35, b) 150, and c) 250 nm NaNbO_3_ films. d–f) Calculated FORC distributions for d) 35, e) 150, and f) 250 nm NaNbO_3_ films revealing a peak from ferroelectric switching across all thicknesses with an addition antiferroelectric‐ferroelectric transition peak in the intermediate 150 nm film.

For confirmation of the role of the AFE phase in the underlying switching mechanism, we performed FORC measurements across the thickness range. For each sample, a positive saturating field (*E_sat_*) was applied to set the polarization state, with the magnitude determined from PUND measurements. Minor hysteresis loops were recorded by ramping down the electric field (*E*) to varying reversal fields (*E_r_)* and then increasing it back to *E_sat_* while measuring the polarization *p(E,E_r_)* (Experimental Section). Selected sequences of minor hysteresis loops obtained from these measurements are shown in **Figure** **3**a–c. FORC distributions are obtained by taking mixed partial derivatives of *p*(*E*, *E_r_*) with respect to *E* and *E_r_* (Figure 3d–f). In connection to the classical Preisach formalism, this can be interpreted as a density distribution of the macroscopic hysteretic system's elementary switchable units, hysterons, each of which is characterized by a rectangular hysteresis loop with a coercive field (*E_c_* = (*E* – *E_r_*)/2) and internal bias field (*E_b_* = (*E* + *E_r_*)/2).^[^ ^45^ ^]^ In Figure 3d–f, the “reversible” switchable units lie along the *E_b_* axis and the “irreversible” switchable units have some component along the *E_c_* axis.

For the 35 and 250 nm samples, we observe results that align with previous work on classical ferroelectric systems,^[^ ^45^, ^46^ ^]^ with the minor hysteresis loops (Figure 3a,c) switching from negative to positive polarization across a narrow range of fields. In the FORC distributions (Figure 3d,f), there is a strong peak along the *E_c_* axis arising from irreversible hysterons associated with ferroelectric switching. This is well separated from a secondary peak from reversible hysterons along the *E_b_* axis. Due to the data collection density and numerical methods for calculating the derivatives, some parts of the reversible peaks are cut off. Additionally, some asymmetry along the *E_b_* axis can be observed, either due to the positive poling of the samples or a preferential polarization for the hysterons near the bottom electrode interface.

FORC measurements performed at an intermediate thickness of 150 nm show notable differences, with the minor hysteresis loops (Figure 3b) displaying a variation in the positive switching fields of different minor hysteresis loops. In the FORC distribution, the ferroelectric peak along the coercive field axis is still present, but there exists an additional irreversible peak at (*E*, *E_r_*) = (225, −676 kV cm^−1^) that signifies a clear deviation from the microscopic switching distributions of a traditional ferroelectric. This peak has large components along both the *E_c_* and *E_b_* axes and can be attributed to the reversible AFE‐to‐FE transition.^[^ ^47^ ^]^ We note that a similar peak near (*E*, *E_r_*) = (676, −225 kV cm^−1^) is expected but could not be measured due to leakage limitations of the sample that prevented application of positive electric fields above 640 kV cm^−1^ for the 150 nm sample during the FORC measurement procedure. Additionally, the asymmetry along the *E_b_* axis observed in the purely ferroelectric samples may also affect the locations of the antiferroelectric‐ferroelectric transition peaks. Regardless, the two peaks in Figure 3e demonstrate that hysterons from both coexisting FE and AFE phases in this intermediate thickness contribute to the film's switching response.

### 2.3 Evaluating Application Potential Across the Thickness Range

**Figure 4:**
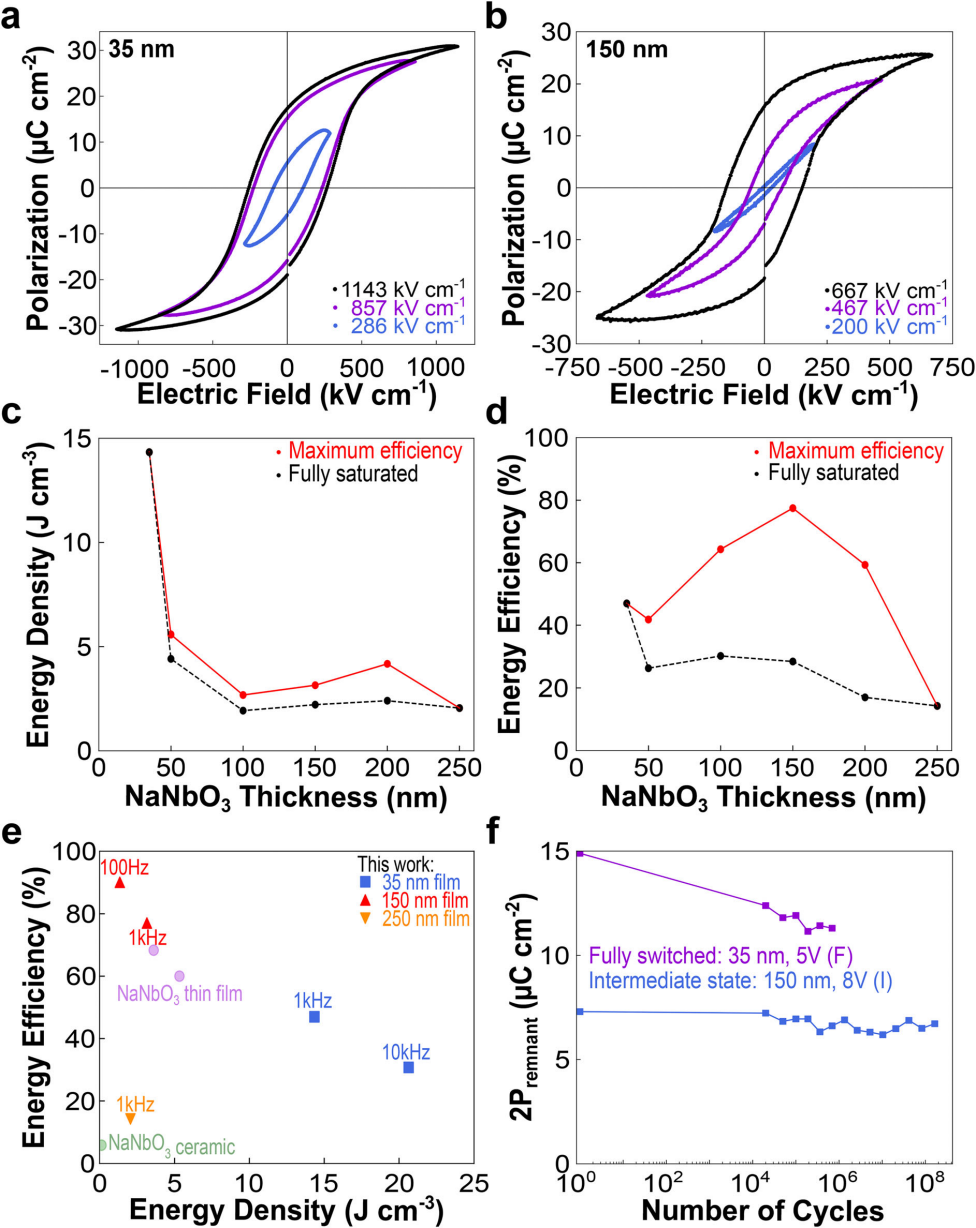
Application potential of NaNbO_3_ thin films. a,b) Applied field dependence of ferroelectric hysteresis loops for (a) 35 nm NaNbO_3_ and (b) 150 nm NaNbO_3_. c,d) Recoverable energy density (c) and energy efficiency (d) as a function of thickness for fully saturated loops (black) and loops achieving maximum efficiency at each thickness through choice of smaller applied field (red), including intermediate switching states (all measured at 1 kHz, corresponding loops and applied electric fields are provided in Figures S12 and S14, Supporting Information). e) Comparison of energy storage properties of various NaNbO_3_ samples including this work as well as literature reports of ceramic NaNbO_3_ (green) and other NaNbO_3_ and NaNbO_3_‐based thin films (purple) (data provided in Table S1, Supporting Information). f) Fatigue measurements for a fully switched polarization state of 35 nm NaNbO_3_ and an intermediate polarization state of 150 nm NaNbO_3_.

The complex switching mechanism arising from the coexisting AFE and FE phases in the intermediate thicknesses leads to a markedly different voltage dependence of the hysteresis loops compared to a classical ferroelectric. In the 35 nm film, the coercive field and remnant polarization remain approximately constant once the applied field exceeds the coercive field of the fully‐switched state (**Figure** **4**a). However, in the 150 nm film, an applied field dependence of the coercive field and remnant polarization persists even once the applied field exceeds the coercive field of the fully‐switched state (Figure 4b). This is due to the disruption of the long‐range order of the FE phase by the AFE phase when the switching remains within the intermediate state, leading to a lower coercive field and remnant polarization than in the fully‐switched state.^[^ ^48^, ^49^ ^]^

From these thickness‐ and voltage‐dependent hysteresis loops, we identify two distinct pathways for size effects in strained NaNbO_3_ films to enhance energy storage properties relative to the bulk. First, the recoverable energy density is dramatically enhanced by reducing thickness, as seen in Figure 4c, with the 35 nm film outperforming the 250 nm film at 1 kHz by more than five times while offering a modest boost in energy efficiency (Figure 4d). Second, the energy efficiency at intermediate thickness films can be significantly improved by leveraging the pinched and narrow hysteresis loops from the thickness‐ and strain‐stabilized AFE‐FE phase coexistence (Figures S12 and S14, Supporting Information) through reduction of the maximum applied field. This enables a nearly threefold increase in the energy efficiency at 150 nm compared to the fully switched state (Figure 4d) and is accompanied by a small improvement in recoverable energy density (Figure 4c) as well as a significant reduction in coercive field leading to a deviation from Janovec‐Kay‐Dunn‐like behavior^[^ ^50^ ^]^ (Figure S15, Supporting Information).

Altering the frequency enables even further improvements, with the 35 nm film surpassing a recoverable energy density of 20 J cm^−3^ at 10 kHz and the intermediate state in the 150 nm film exhibiting an efficiency of 90% at 100 Hz, each comparable to the highest reported values in pure NaNbO_3_ and NaNbO_3_‐based ceramics (Figure 4e; Figure S16, and Table S1, Supporting Information).^[^ ^25^, ^30^, ^51^, ^52^, ^53^, ^54^, ^55^, ^56^, ^57^, ^58^, ^59^, ^60^, ^61^, ^62^, ^63^, ^64^, ^65^, ^66^, ^67^, ^68^, ^69^, ^70^, ^71^, ^72^, ^73^ ^]^ Both routes significantly improve both the recoverable energy density and energy efficiency relative to the poor performance of bulk NaNbO_3_, but not simultaneously in a single film. For practical applications, either reducing thickness or stabilizing an intermediate state from the FE‐AFE phase coexistence could be applied to epitaxial thin films of better performing, chemically‐substituted NaNbO_3_‐based ceramics to maximize both parameters simultaneously.

In intermediate thickness films with multistate switching, the stable intermediate state is also found to be nearly fatigue‐free, despite the films themselves experiencing fatigue when operating at full switching (Figure 4f; Figure S8, Supporting Information). This strong fatigue performance, combined with good retention of both the fully‐switched and intermediate states in Figure 2h, demonstrates the potential for AFE‐FE phase coexistence as a mechanism for generating multiple accessible polarization states for enhanced non‐volatile memories.

## 3 Conclusion

In summary, we demonstrate the effects of thickness scaling under coherent epitaxial strain on the ferroic order and electrical properties of NaNbO_3_ thin films with high crystallinity and low leakage currents. Increasing thickness initially leads to a transition from a single ferroelectric phase to a coexistence of ferroelectric and antiferroelectric phases that displays multistate switching. The intermediate switching state is found to enable multiple accessible remnant polarizations with strong retention and fatigue performance. With a further thickness increase, we find a mixed orientation structure that appears as a strain relief mechanism and suppresses antiferroelectricity, leading to purely ferroelectric behavior in the 250 nm thick film. Evaluating the energy storage properties over the thickness range reveals two pathways by which these strained thin films improve the properties of bulk NaNbO_3_: improving the energy storage density through miniaturization (crossing 20 J cm^−3^ for 35 nm NaNbO_3_) and improving the energy efficiency using the complex switching mechanism from the ferroelectric‐antiferroelectric phase coexistence (reaching up to 90% at 150 nm). Future work could apply such techniques to further enhance the improvements in chemically substituted antiferroelectric systems that optimize bulk performance.

## 4 Experimental Section

### Thin Film Synthesis

Tri‐layer epitaxial heterostructures of La_0.7_Sr_0.3_MnO_3_ (10 nm thick) / NaNbO_3_ (35‐250 nm thick) / La_0.7_Sr_0.3_MnO_3_ (25 nm thick) were synthesized on (001)‐oriented single‐crystalline SrTiO_3_ substrates via pulsed laser deposition in a single chamber with a KrF excimer laser (λ = 248 nm). The polycrystalline non‐stoichiometric Na_1.2_NbO_3_ target was prepared by grinding and pressing a mixture of Na_2_CO_3_ and Nb_2_O_5_, decarbonating at 950 °C for 6 hr, then subsequently regrinding, repressing, and sintering at 950 °C for 12 hr. The La_0.7_Sr_0.3_MnO_3_ layers were grown with an oxygen pressure of 200–220 mTorr, a laser fluence of 1.52 J cm^−2^, an imaged laser spot size of 4.93 mm^2^, and a repetition rate of 3 Hz, with the bottom 10 nm layer grown at 700 °C and the top 25 nm layer grown at 600 °C. The NaNbO_3_ layer was grown at 600 °C with an oxygen pressure of 300–330 mTorr, a laser fluence of 1.82 J cm^−2^, an imaged laser spot size of 6.1 mm^2^, and a repetition rate of 2 Hz. After growth, the chamber was cooled down to room temperature in 1.5 torr O_2_ at a cooling rate of 5 °C min^−1^.

### Structural and Surface Characterization

The *θ*–2*θ* X‐ray diffraction scans and 2D reciprocal space maps were measured using an Empyrean diffractometer (Malvern Panalytical) with a monochromatic Cu‐Kα1 source (1.540598 Å). Piezoresponse force microscopy (PFM) measurements were taken using a Cypher AFM (Asylum Research) in Vector PFM mode using Ir/Pt‐coated conductive tips with a force constant of ≈2.8 N m^−1^ (Nanosensor, PPP‐EFM). All PFM measurements were collected with the PFM tip aligned along the [110] direction of the substrate.

### Device Fabrication

All electrical properties were measured using symmetric circular capacitors, with the voltage applied to the top electrode and the bottom electrode connected to ground. The 100‐micron diameter circular top electrodes were defined by photolithographically patterning AZ 1512 photoresist with 1.2 µm thickness using a Durham Magneto Optics ML3 MicroWriter direct write machine and subsequent acid etching of the top La_0.7_Sr_0.3_MnO_3_ layer in a 1:4 H_3_PO_4_ (14.8 m) to water solution. Contact was made with the bottom La_0.7_Sr_0.3_MnO_3_ electrode using silver paint after scratching the tri‐layer structure with a diamond pen.

### Leakage Measurements

Leakage currents were performed with a 2400 SourceMeter unit (Keithley) by collecting current – applied voltage curves at room temperature in ambient conditions. The applied voltage was increased at 100 mV s^−1^ to the maximum voltage (either 3 V or 8 V), held for 5 s, then swept to an equivalent negative voltage at the same rate, held for 5 s, and returned to 0 V. For leakage current optimization, 10–16 randomly selected capacitors were measured from each sample to determine the optimal synthesis conditions.

### Ferroelectric and Dielectric Property Measurements

Ferroelectric properties were measured using a Precision Multiferroic tester (Radiant Technologies) at room temperature in ambient conditions. PUND measurements were performed using the pulse train in Figure S8a (Supporting Information). An initial negative preset pulse was applied to set the polarization state, followed by a pulse delay for the polarization to return to its negative remnant value. This was then followed by a positive switching pulse to measure the full switched polarization (P*), an additional delay to return to the positive remnant polarization, and a second positive pulse to measure the unswitched polarization (*P^*). By subtracting the unswitched polarization from the switched polarization, information regarding the remnant polarization of the film was obtained: *2P_remnant_* = P* – P^. For all voltage‐dependent PUND measurements, the applied voltage was increased in 100 mV increments with a 1 ms pulse delay and constant pulse width of 1 ms. The pulse width was kept constant at 1 ms for Figure 2e,f and Figure S10 (Supporting Information), and was varied between 10 µs, 100 µs, and 1 ms in Figure S11 (Supporting Information). Retention measurements were taken using the pulse train in Figure S8b (Supporting Information) at a 10 µs pulse width. Dielectric measurements were taken using an E4980A LCR meter (Agilent Technologies) on poled capacitor structures with 8.6 kV cm^−1^ AC field and 0 V DC bias.

In fatigue measurements, PUND measurements were taken before and after the sample was repeatedly cycled by a switching waveform, as depicted in Figure S8c (Supporting Information). The switching waveform was a square wave applied at 10 kHz and the PUND measurements were taken with a 1 ms pulse delay and 100 µs pulse width. The maximum voltage applied for both the switching waveform and the PUND measurement was 5 V for the 35 nm NaNbO_3_ film and 8 V for the 150 nm NaNbO_3_ film.

FORC measurements were recorded using a positive saturating field whose magnitude depended on the sample thickness. The field was lowered to varying reversal fields and then returned to the saturating field at a frequency of 1 kHz while recording the polarization. The polarization *p*(*E*, *E_r_*) during the ascending return sweep was used to calculate the FORC distribution *ρ*(*E*, *E_r_*) by taking mixed second derivatives with respect to both *E* and *E_r_*:

(1) $$\begin{array}{c} \rho \;\left(E,E_{r}\right)=\frac{1}{2}\;\frac{\partial^{2}p\left(E,E_{r}\right)}{\partial E\partial E_{r}} \end{array}$$

Energy storage parameters were extracted from the polarization – electric field hysteresis curves by numerically integrating the positive electric field data. Recoverable energy density is given by:

(2) $$\begin{array}{c} W_{rec}=\int_{P_{rem}}^{P_{sat}}E_{descend}dP \end{array}$$

integrating along the descending branch of the hysteresis curve, while *W_loss_* is the area inside the hysteresis curve given by:

(3) $$\begin{array}{c} W_{\mathrm{loss}}=\int_{0}^{P_{\mathrm{sat}}}E_{\mathrm{asce}\mathrm{nd}}\mathrm{dP}-W_{\mathrm{rec}} \end{array}$$

From this, calculate the energy storage efficiency:

(4) $$\begin{array}{c} \eta =\frac{W_{rec}}{W_{rec}+W_{loss}}\times 100\% \end{array}$$

## Conflict of Interest

The authors declare no conflict of interest.

## Supporting information



Supporting Information

## Data Availability

The data that support the findings of this study are available from the corresponding author upon reasonable request.
