# Supplementary material for: Coexisting Phases in NaNbO3 Thin Films Influenced by Epitaxial Strain and Size Effects
Source: Adv Sci (Weinh). 2025 Oct 14;12(46):e10099. doi: 10.1002/advs.202510099 (PMC12697832; doi:10.1002/advs.202510099)
Supplement: Supplementary file 1 — Supporting Information [file ADVS-12-e10099-s001.docx]

Supporting Information

Coexisting Phases in NaNbO_3_ Thin Films Influenced by Epitaxial Strain and Size Effects

Aarushi Khandelwal, Kevin J. Crust*, Reza Ghanbari, Yijun Yu, Ruijuan Xu*, and Harold Y. Hwang

**Figure S1**

**
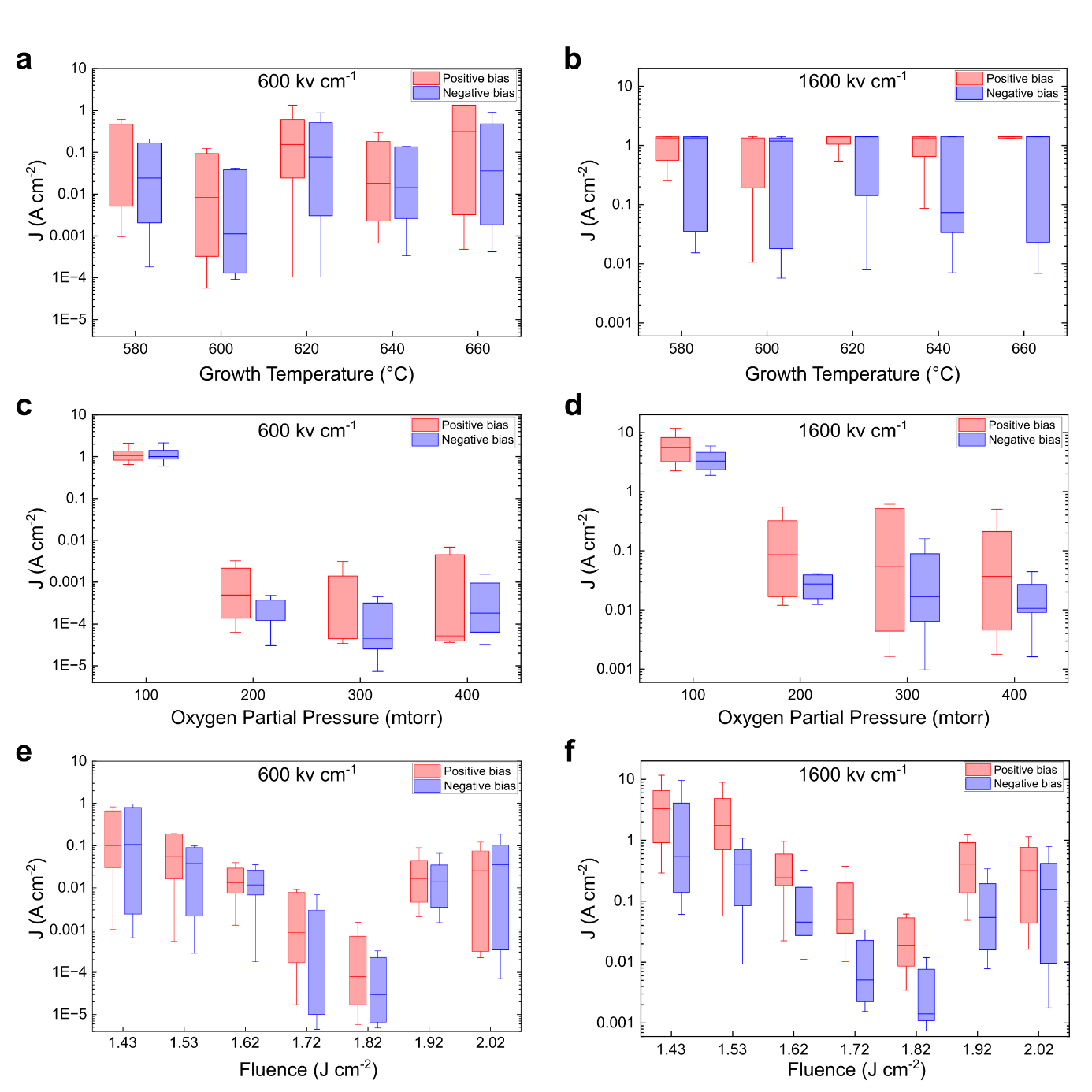
**

**Figure S1.** Leakage current optimization. Box charts displaying the distributions of current densities measured from randomly selected capacitors in La_0.7_Sr_0.3_MnO_3_ / NaNbO_3_ / La_0.7_Sr_0.3_MnO_3_ / SrTiO_3_ heterostructures. The (a,b) temperature, (c,d) oxygen partial pressure, and (e,f) fluence of the 50 nm NaNbO_3_ layer were varied to determine the optimal synthesis conditions. Leakage currents were extracted from current – voltage (IV) measurements at maximum electric fields of (a,c,e) 600 kV cm^-1^ and (b,d,f) 1600 kV cm^-1^. The box plots the lower quartile, median, and upper quartile, while the upper (lower) whisker plots the outermost data point within the 75^th^ percentile + 1.5 * interquartile range (25^th^ percentile – 1.5 * interquartile range).

**Figure S2**

**
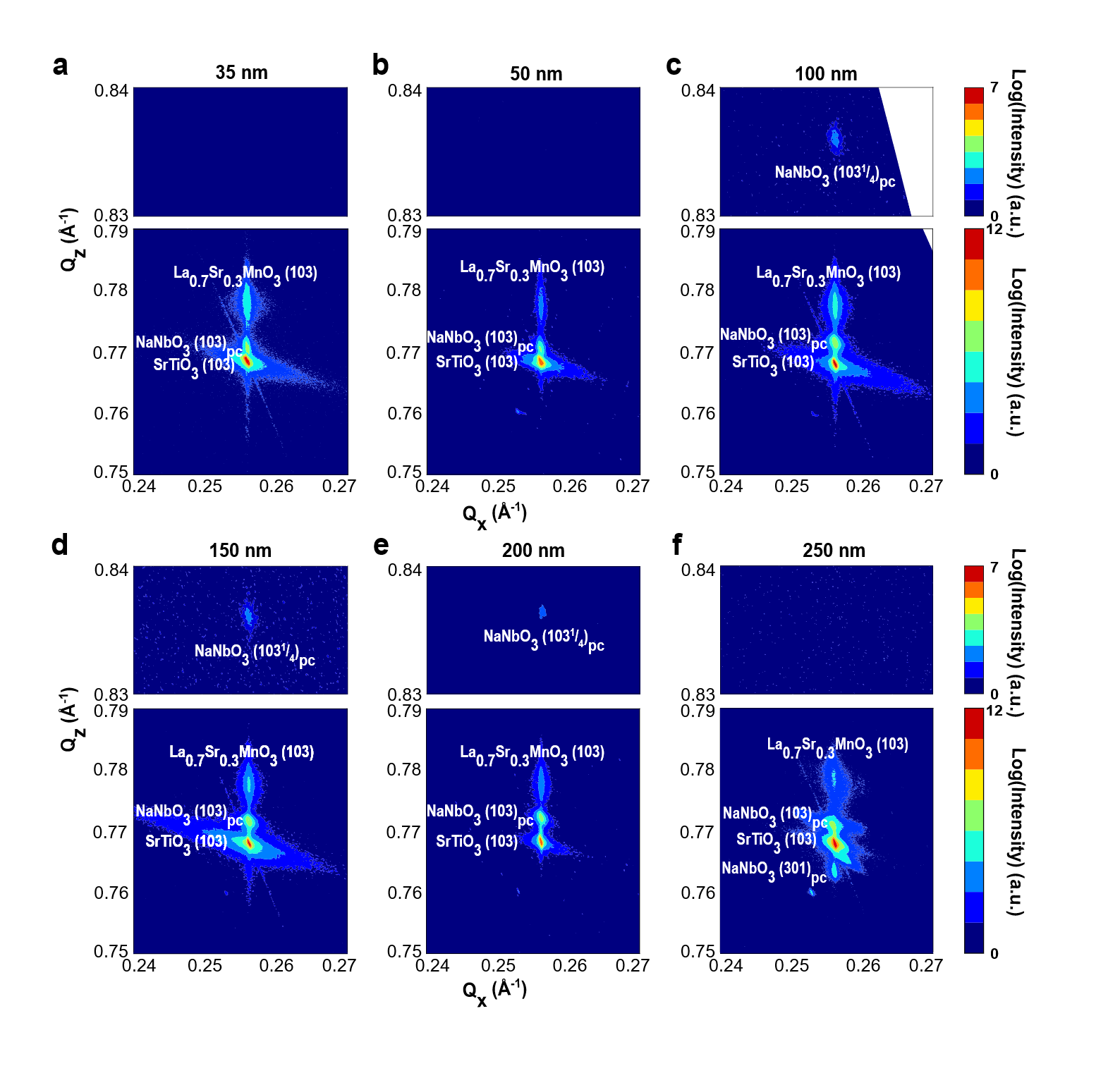
**

**Figure S2.** Thickness dependence of film orientation and phase from structural characterization. X-ray reciprocal space maps about the SrTiO_3_ (103) diffraction condition for heterostructures with (a) 35, (b) 50, (c) 100, (d) 150, (e) 200, and (f) 250 nm thick NaNbO_3_.

**Figure S3**


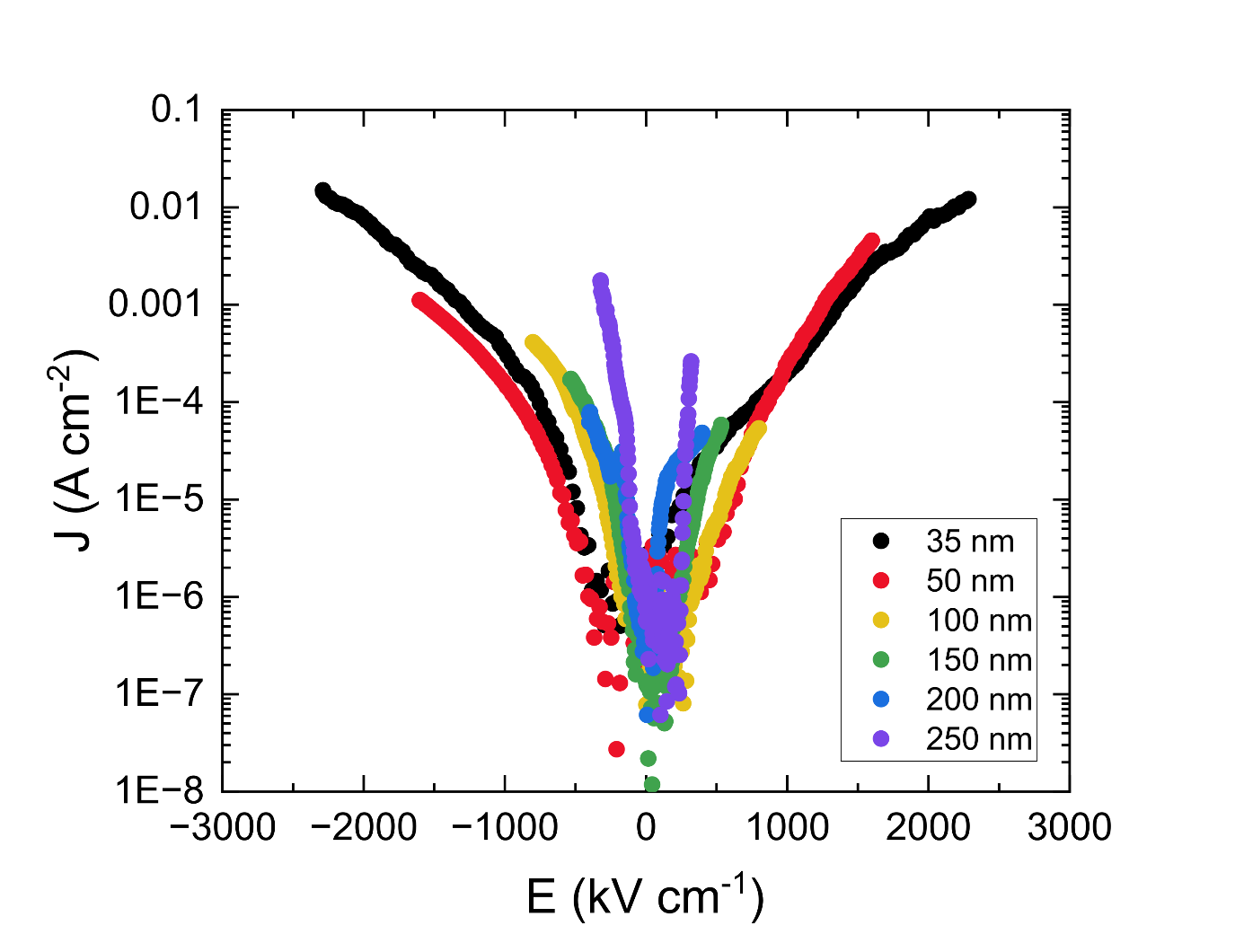


**Figure S3.** Thickness dependence of leakage current. Current – voltage measurements, converted into current density – electric field, of the La_0.7_Sr_0.3_MnO_3_ / NaNbO_3_ / La_0.7_Sr_0.3_MnO_3_ capacitors with varying NaNbO_3_ film thickness. A maximum of 8 V was applied to each capacitor, leading to a reduction in the maximum applied field with increasing thickness.

**Figure S4**


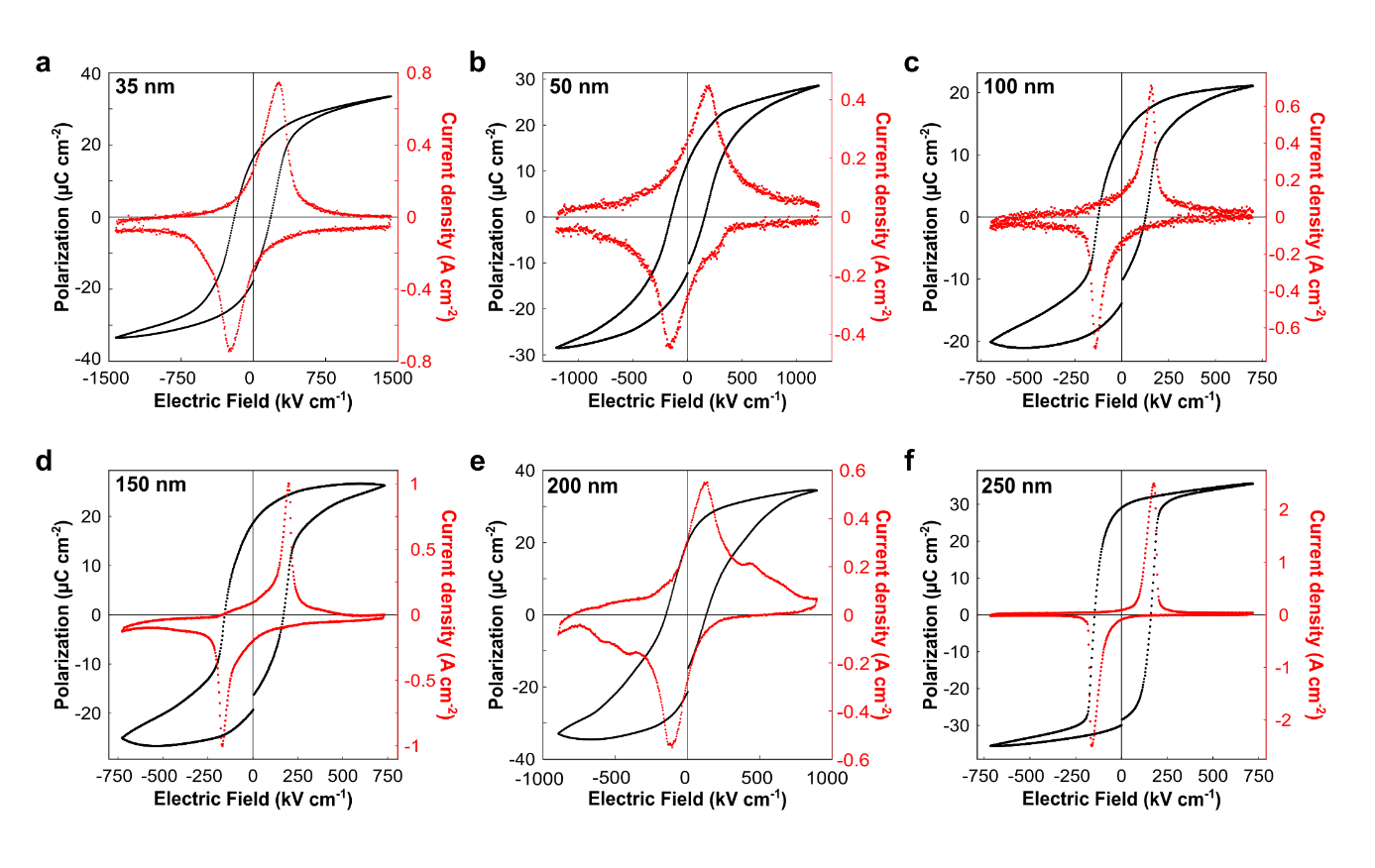


**Figure S4.** Thickness dependence of ferroelectric hysteresis loops. Polarization – electric field hysteresis measurements and corresponding switching current – electric field hysteresis measurements at 1 kHz for (a) 35, (b) 50, (c) 100, (d) 150, (e) 200, and (f) 250 nm thick NaNbO_3_.

**Figure S5**


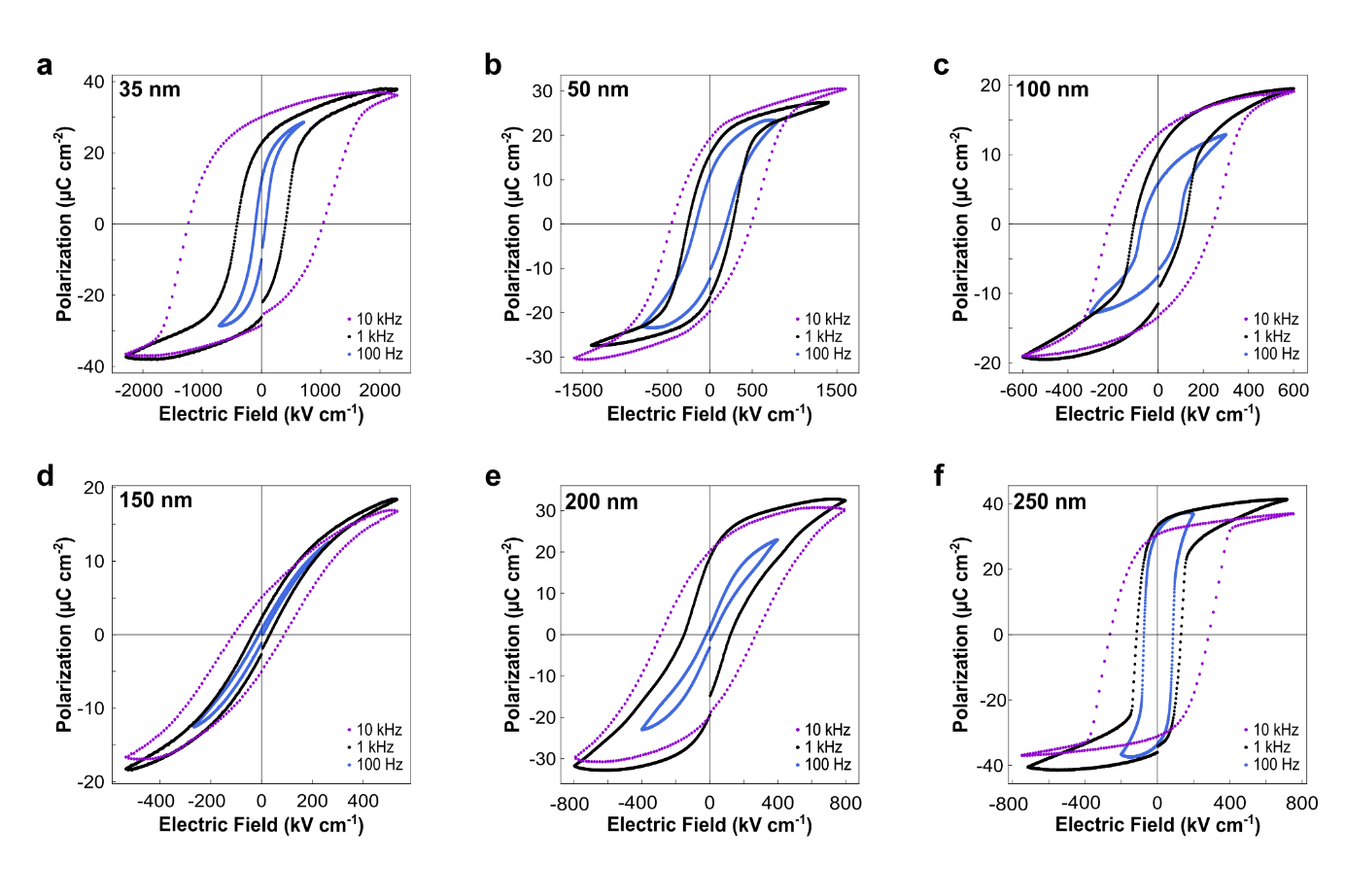


**Figure S5.** Frequency dependence of ferroelectric hysteresis. Polarization – electric field hysteresis loops for (a) 35, (b) 50, (c) 100, (d) 150, (e) 200, and (f) 250 nm thick NaNbO_3_.

**Figure S6**


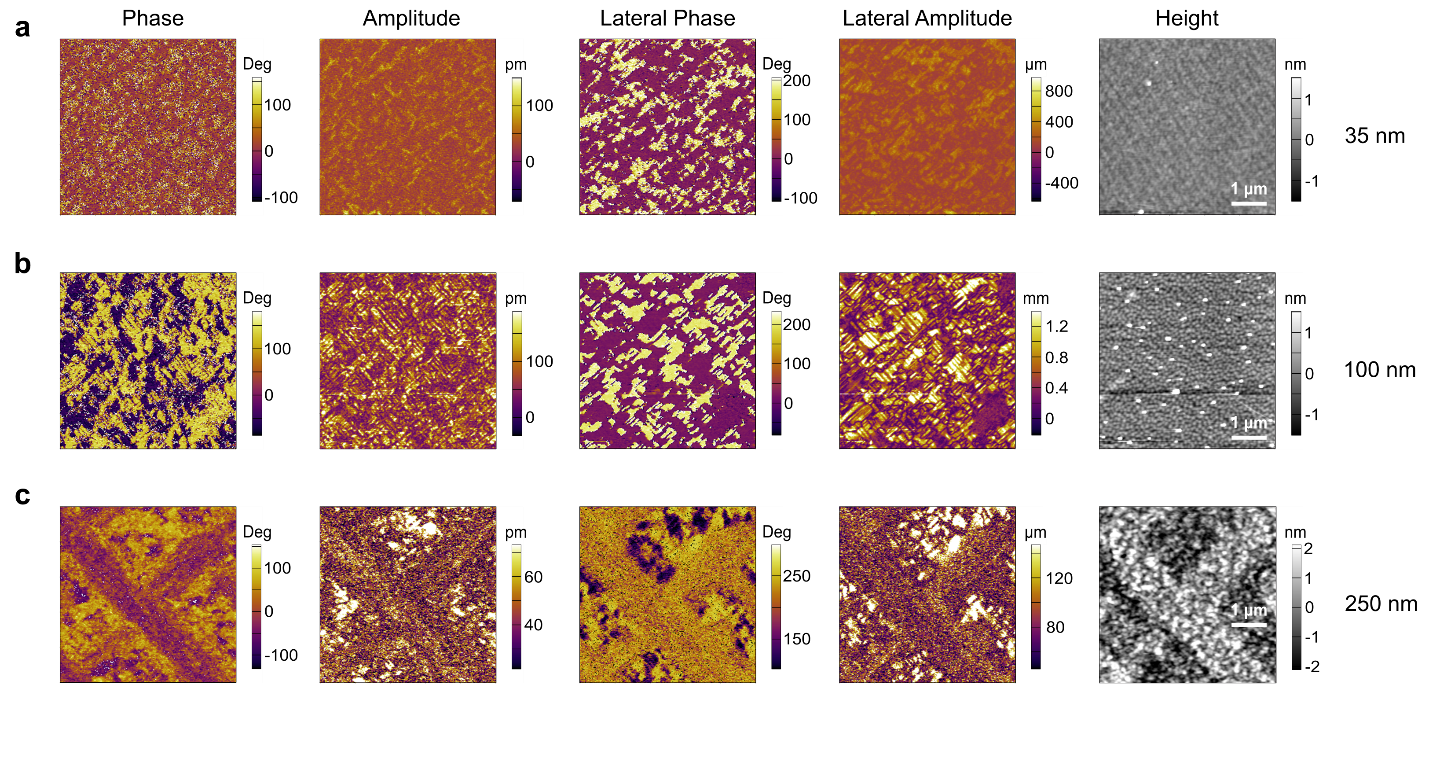


**Figure S6.** Thickness dependence of piezoresponse force microscopy. Vertical phase, vertical amplitude, lateral phase, lateral amplitude, and topography piezoresponse force microscopy (PFM) images of NaNbO_3_ / La_0.7_Sr_0.3_MnO_3_ / SrTiO_3_ heterostructures with various thicknesses of NaNbO_3_. The (a) 35 nm and (b) 100 nm samples show atomically smooth surfaces with clear domain patterns and stronger in-plane polarization. The (c) 250 nm sample shows laterally segregated regions with different heights, which is attributed to the separate peaks observed in the X-ray structural characterization. The lower region continues to show stronger in-plane polarization with clear domains, while the higher region shows a relatively stronger out-of-plane polarization signal. Images are collected with the PFM tip aligned along the [110] direction of the substrate.

**Figure S7**


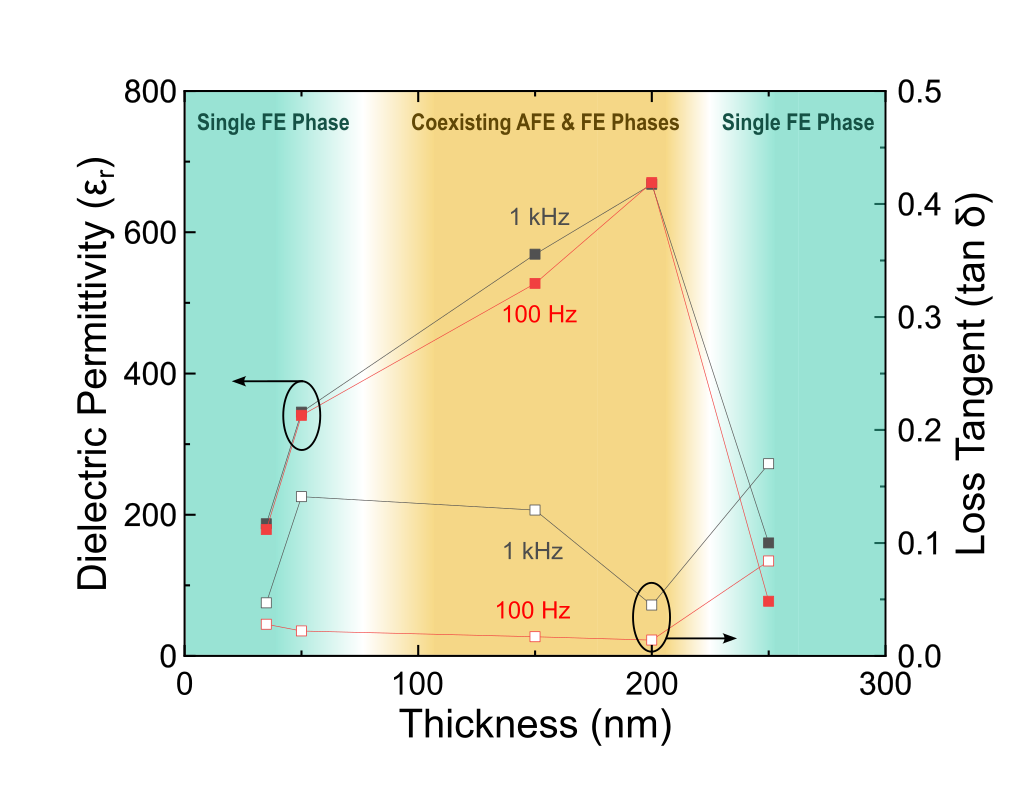


**Figure S7.** Thickness dependence of dielectric permittivity and loss tangent at 1 kHz and 100 Hz. Relative dielectric permittivity and loss tangent of poled NaNbO_3_ at various thicknesses, measured at 0 V DC bias. The permittivity is significantly increased in the thicknesses where an antiferroelectric quarter-order superlattice reflection is observed in the reciprocal space maps, as is expected for a material with multiple ferroic orders near a phase boundary.

**Figure S8**

**
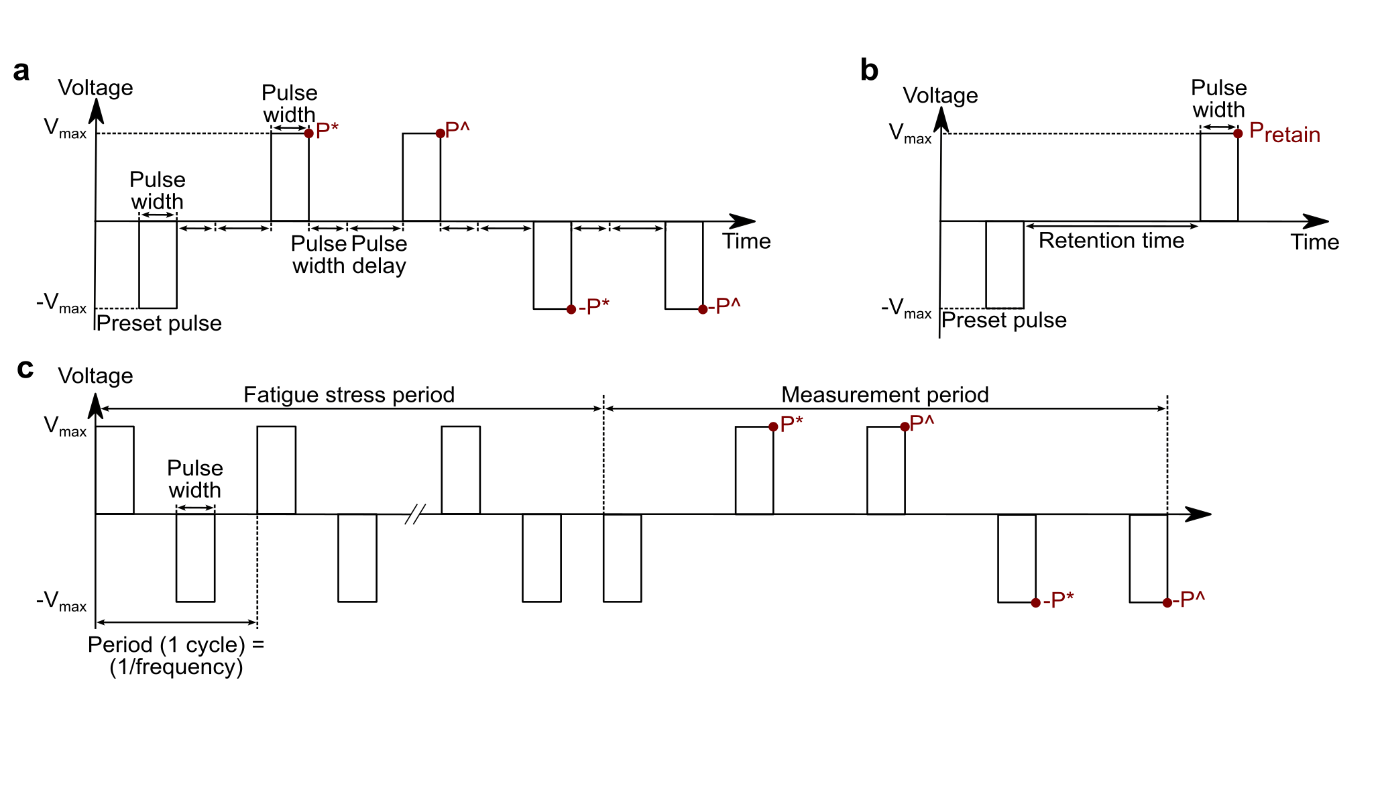
**

**Figure S8.** Pulse sequences of electrical measurements. Pulse sequences for the (a) PUND, (b) retention, and (c) fatigue measurements. For PUND and fatigue measurements, both the switched polarization (P*) and unswitched polarization (P^) polarizations were measured, from which the remnant polarization can be calculated: 2*P_remnant_* = *P** - *P^*.

**Figure S9**


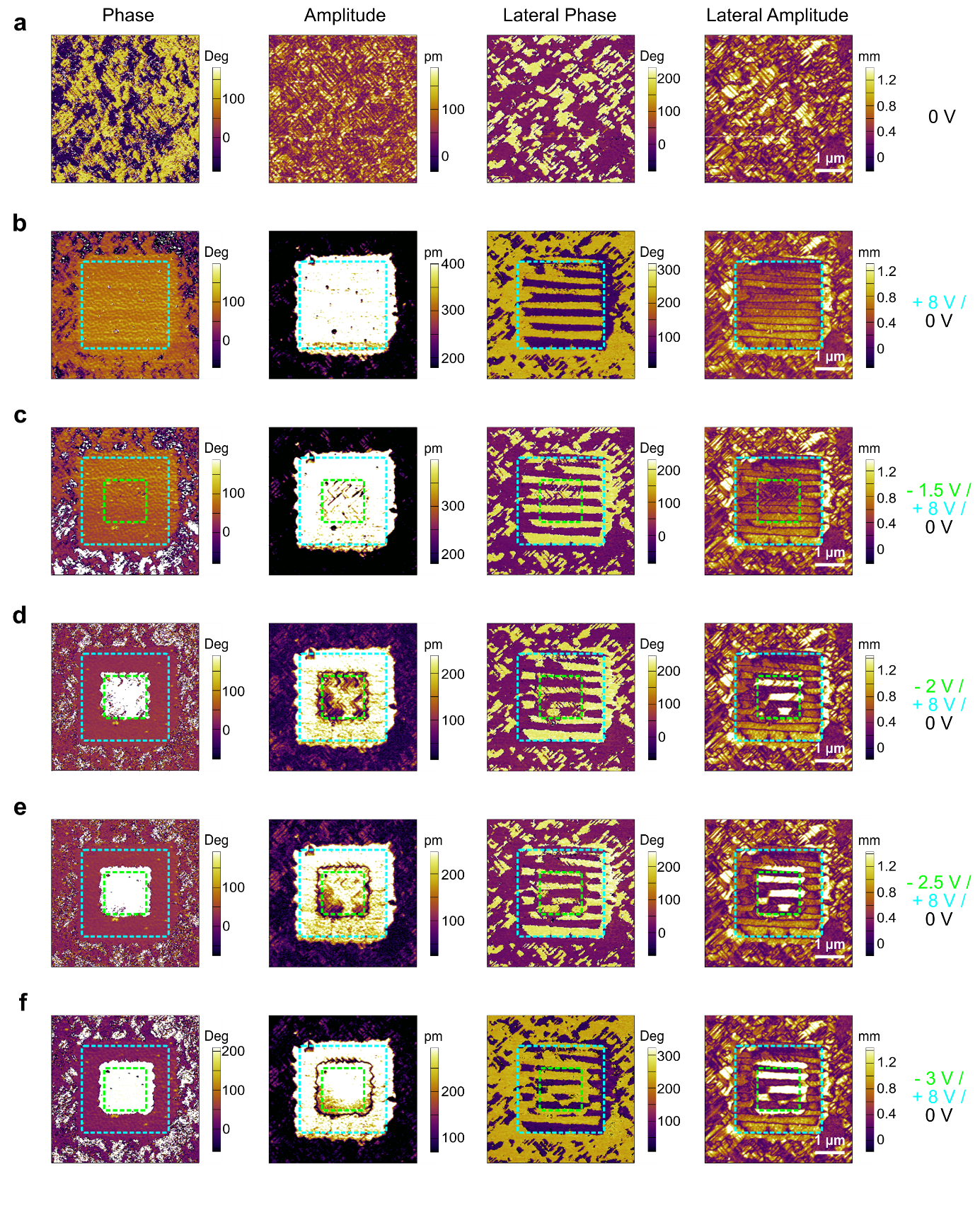


**Figure S9.** Piezoresponse force microscopy of 100 nm NaNbO_3_ switching. Vertical phase, vertical amplitude, lateral phase, and lateral amplitude piezoresponse force microscopy (PFM) images taken after box-in-box poling of a NaNbO_3_ / La_0.7_Sr_0.3_MnO_3_ / SrTiO_3_ heterostructures with 100 nm NaNbO_3_. +8 V was first applied (b) to a 3 x 3 µm^2^ area (blue), with subsequent increasing negative voltages applied (c-f) to an inner 1.5 x 1.5 µm^2^ area (green).

**Figure S10**


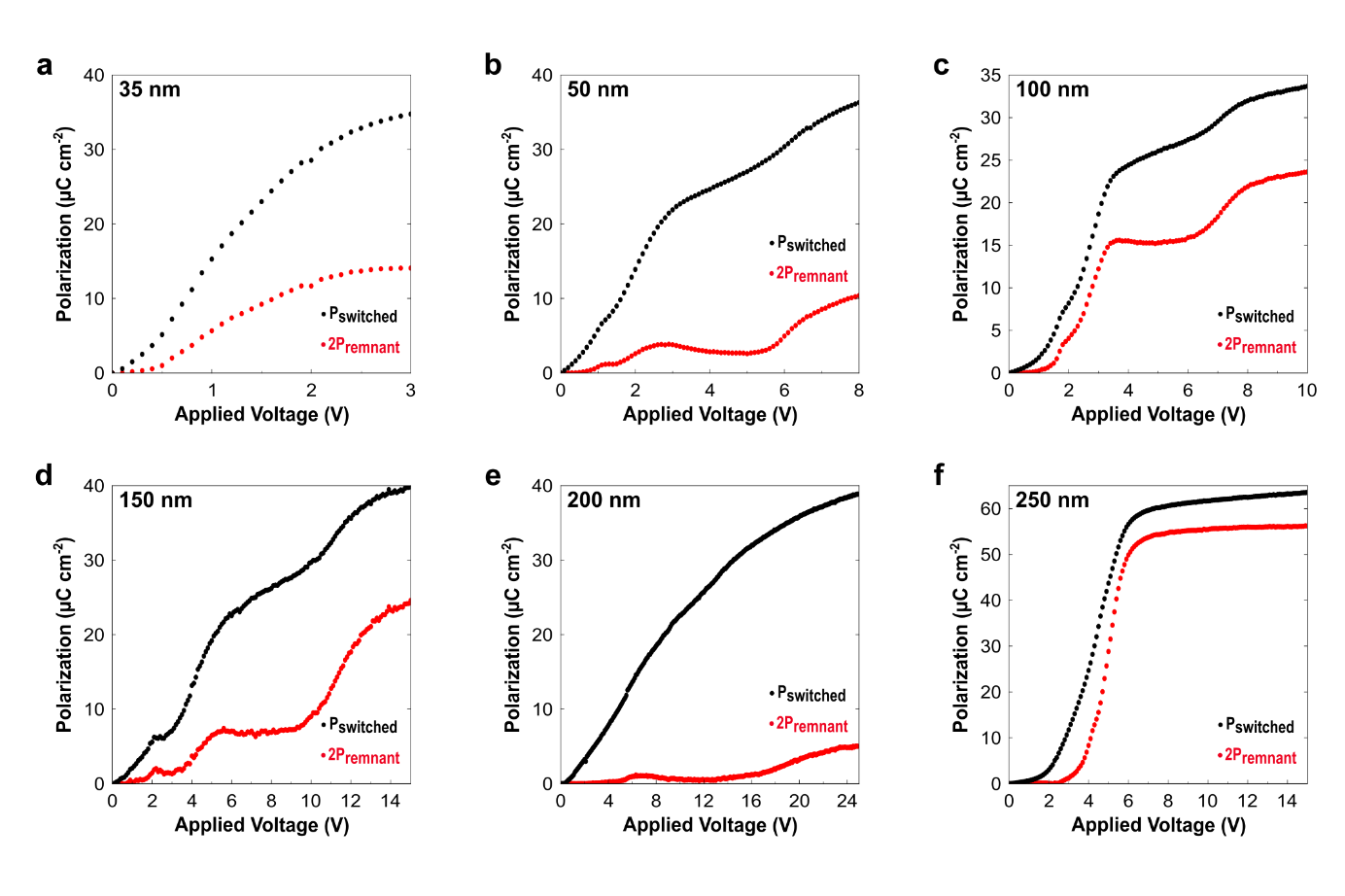


**Figure S10.** Thickness dependence of PUND. Evolution of switched polarization and remnant polarization from PUND measurements as a function of applied voltage with a 1ms pulse width for (a) 35, (b) 50, (c) 100, (d) 150, (e) 200, and (f) 250 nm thick NaNbO_3_.

**Figure S11**


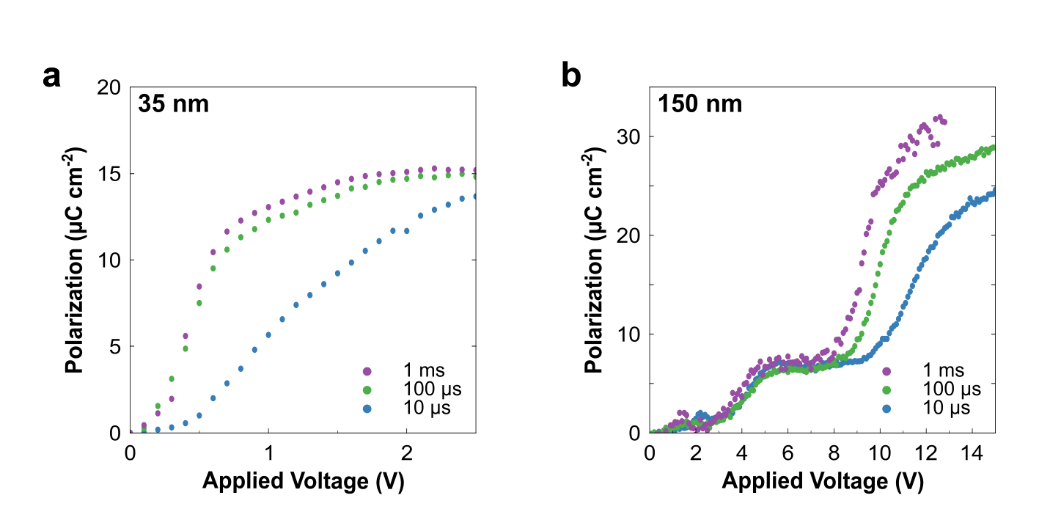


**Figure S11.** Pulse width dependence of PUND. PUND measurements of the remnant polarization as a function of applied voltage for (a) 35 and (b) 150 nm thick NaNbO_3_ with varying pulse widths.

**Figure S12**


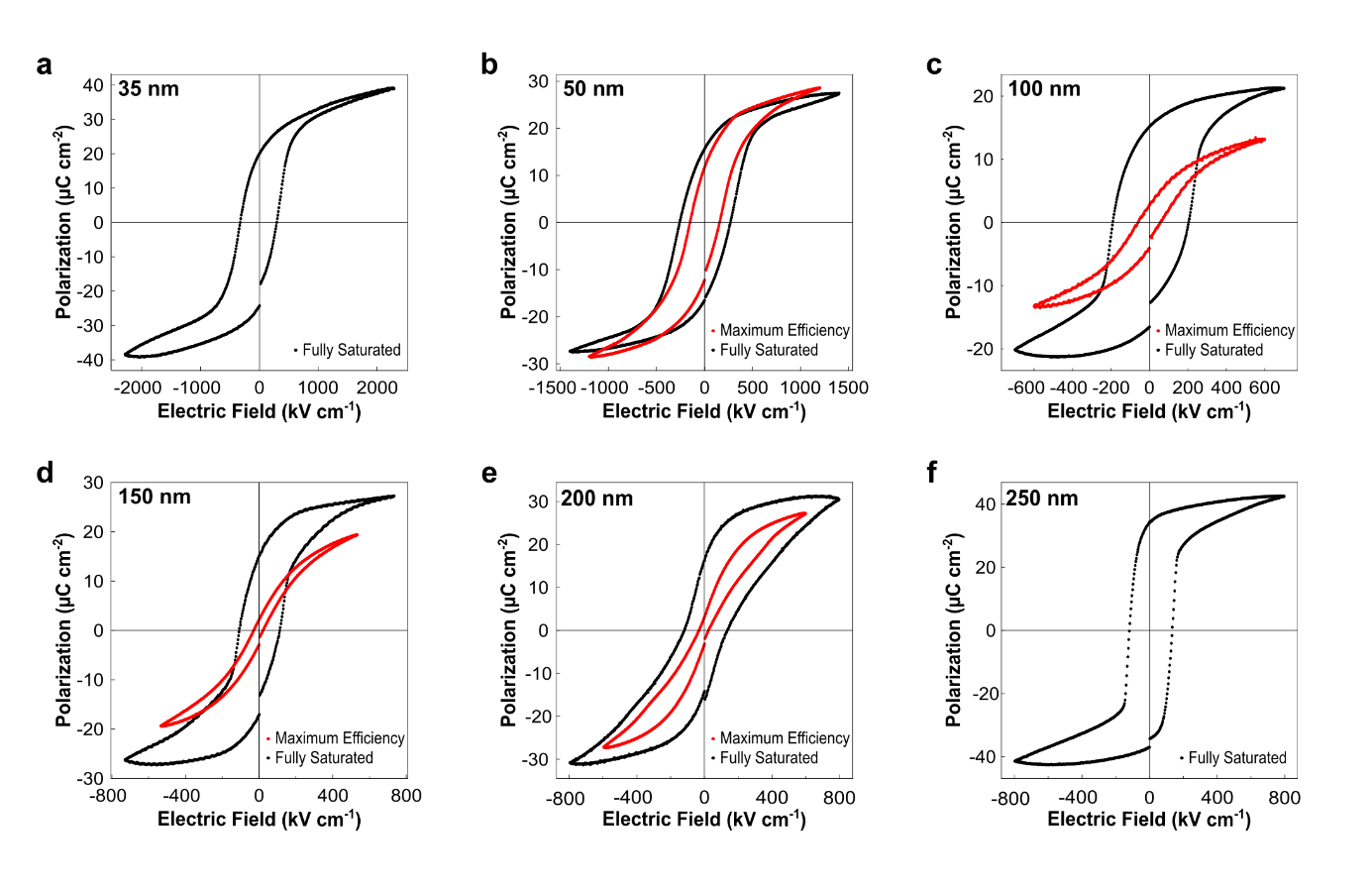


**Figure S12.** Variation of ferroelectric hysteresis for maximum efficiency. Polarization – electric field measurements at 1 kHz for (black) full switching, with the applied field necessary for complete saturation, and (red) the applied field achieving highest efficiency at the given thickness with NaNbO_3_ thicknessess of (a) 35 nm, (b) 50 nm, (c) 100 nm, (d) 150 nm, (e) 200 nm, and (f) 250 nm.

**Figure S13**

**
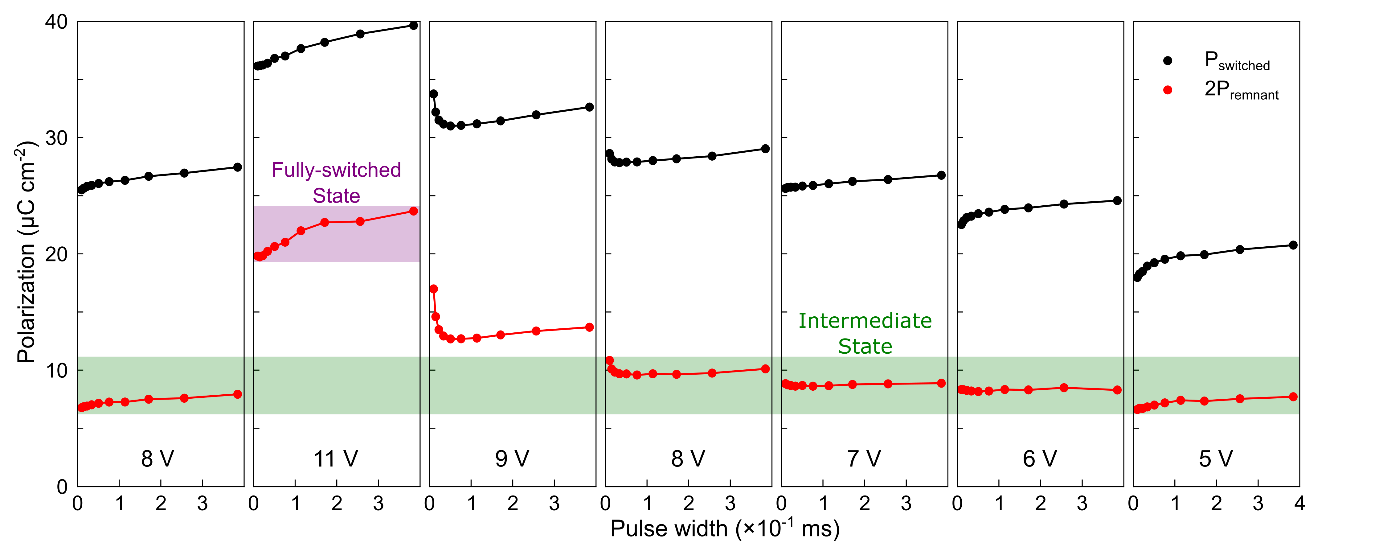
**

**Figure S13.** Reversibility of multiple polarization states in PUND measurements in 150 nm NaNbO_3_. Successive PUND measurements (pulse sequence in Figure S8) were performed with varying voltage (in the left-to-right order shown in the figure), with steadily increasing pulse width utilized at each voltage. Both the switched and remnant polarizations indicate a return to the intermediate state upon application of an intermediate voltage (5-8 V) even after the sample is poled to the fully-switched state (11 V).

**Figure S14**


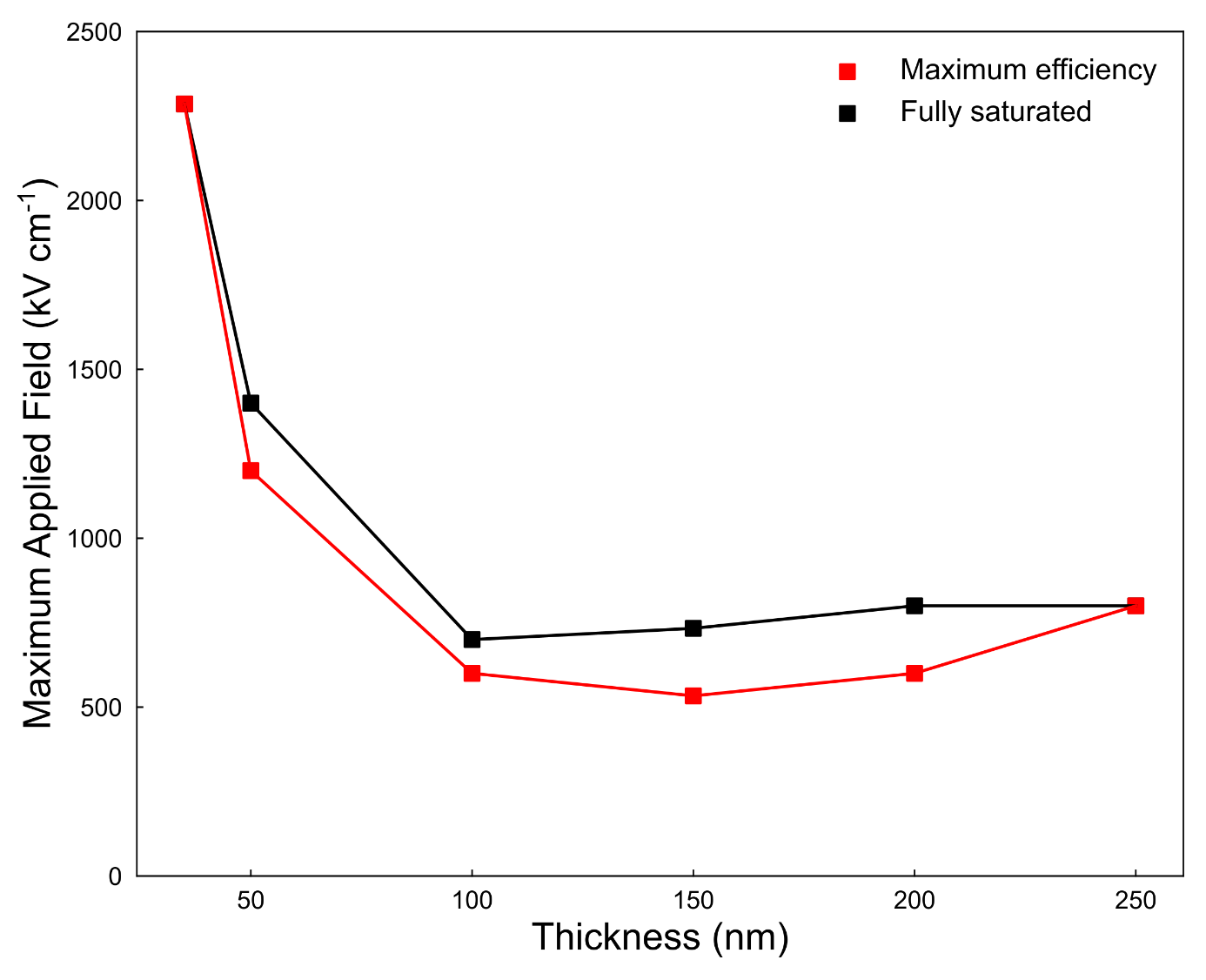


**Figure S14.** Required maximum applied electric fields for full saturation and maximum efficiency corresponding to the reported loops in Figure S11 at 1 kHz. (black) Fully switched loops with the field required to reach full saturation and (red) loops with highest efficiency at that thickness, including intermediate switching states.

**Figure S15**


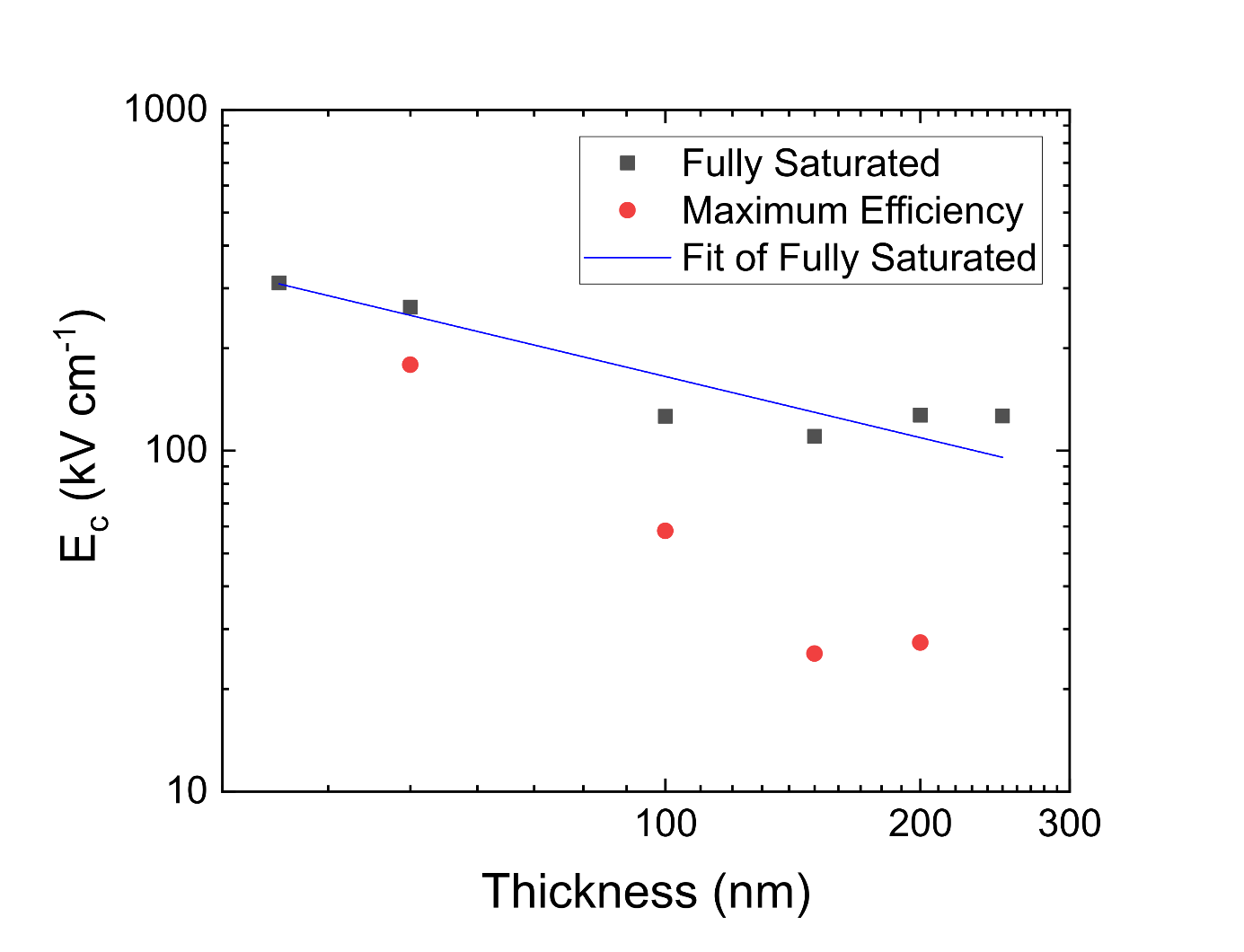


**Figure S15.** Deviation from Janovec-Kay-Dunn-like behavior for maximum efficiency loops. Coercive field as a function of thickness for the fully saturated and maximum efficiency hysteresis loops from Figure S11. The fully saturated loops closely follow Janovec-Kay-Dunn-like scaling, with a power law (y = a*x^b) fit yielding parameters of a = 2571.17172 ± 1044.04016 and b = -0.59604 ± 0.09867. The predicted exponent of -2/3 from the semi-empirical scaling relationship is within the error bounds. When operating at lower maximum applied fields to achieve maximum efficiency, the coercive field no longer follows the same scaling relationship.

**Figure S16**


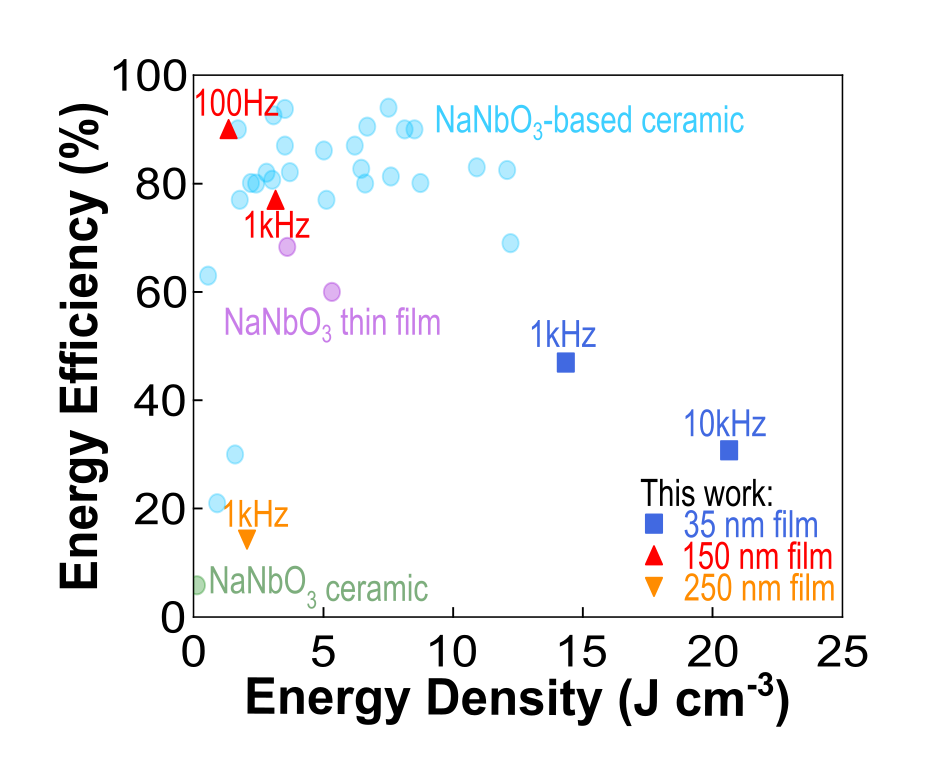


**Figure S16.** Comparison of energy storage parameters with NaNbO_3_-based ceramics. Energy storage properties of various NaNbO_3_-based systems, including this work as well as literature reports of pure ceramic NaNbO_3_ (green), NaNbO_3_-based ceramic compositions (light blue), and other NaNbO_3_ and NaNbO_3_-based thin films (purple) (data provided in Table S1). The intermediate state enables efficiencies comparable chemically-doped ceramics while the thinnest 35 nm film exceeds any previously reported energy storage densities for NaNbO_3_-based systems.

**Table S1.** Energy storage parameters of NaNbO_3_ and NaNbO_3_-based materials from previous reports in literature with accompanying references.

| Reference | Composition | Sample Geometry | Energy Density  [J cm^-3^] | Energy Efficiency  [%] |
| --- | --- | --- | --- | --- |
| M.-H. Zhang et al. (2023) ^[30]^ | NaNbO_3_ | Bulk | 0.12 | 5 |
| H. Qi et al. (2019)^[51]^ | 0.76NaNbO_3_ – 0.24(Bi_0.5_Na_0.5_)TiO_3_ | Bulk | 12.2 | 69 |
| M.-H. Zhang et al. (2023)^[30]^ | 0.95NaNbO_3_ – 0.05SrSnO_3_ | Bulk | 0.9 | 21 |
| M.-H. Zhang et al. (2023)^[30]^ | 0.91NaNbO_3_ – 0.09SrSnO_3_ + 1% wt. MnO_2_ | Bulk | 1.7 | 90 |
| Z. Chen et al. (2022) ^[52]^ | 0.5NaNbO_3_ – 0.5NaTaO_3_ | Bulk | 2.2 | 80.1 |
| Z. Liu et al. (2018) ^[53]^ | 0.96NaNbO_3_ – 0.04CaZrO_3_ | Bulk | 0.55 | 63 |
| A. Xie et al. (2022) ^[54]^ | 0.84NaNbO_3_ – 0.16CaTiO_3_ | Bulk | 6.6 | 80 |
| A. Xie et al. (2022) ^[54]^ | 0.82NaNbO_3_ – 0.18CaTiO_3_ | Bulk | 6.2 | 87 |
| H. Qi et al. (2020) ^[55]^ | 0.57BiFeO_3_ – 0.33BaTiO_3_ – 0.1NaNbO_3_ + 0.1% wt. MnO_3_ + 0.2% wt BaCu(B_2_O_5_) | Bulk | 8.12 | 90 |
| J. Lei et al. (2024) ^[56]^ | 0.82NaNbO_3_ – 0.1BaZrO_3_ – 0.08Bi(Mg_0.5_Ti_0.5_)O_3_ | Bulk | 7.5 | 94 |
| S. Zhang et al. (2024) ^[57]^ | 0.8NaNbO_3_ – 0.2Bi(Ni_0.5_Hf_0.5_)O_3_ | Bulk | 6.45 | 82.72 |
| C. Sun et al. (2021) ^[58]^ | 0.78NaNbO_3_ – 0.22Ba(Mg_1/3_Nb_2/3_)O_3_ | Bulk | 3.51 | 87 |
| H. Qi et al. (2019) ^[59]^ | 0.9NaNbO_3_ – 0.06BaZrO_3_ – 0.04CaZrO_3_ | Bulk | 1.59 | 30 |
| J. Jiang et al. (2021) ^[60]^ | (Na_0.91_Bi_0.09_)(Nb_0.94_Mg_0.06_)O_3_ | Bulk | 10.9 | 83 |
| S. Zhang et al. (2023) ^[61]^ | 0.8NaNbO_3_ – 0.2Bi(Mg_0.5_Hf_0.5_)O_3_ | Bulk | 3.51 | 93.77 |
| A. Xie et al. (2024) ^[62]^ | 0.68NaNbO_3_ – 0.32(Bi_0.5_Li_0.5_)(Zr_0.4_Ti_0.6_)O_3_ | Bulk | 8.5 | 90 |
| A. Xie et al. (2021) ^[63]^ | 0.68NaNbO_3_ – 0.32(Bi_0.5_Li_0.5_)TiO_3_ | Bulk | 8.73 | 80.1 |
| J. Ye et al. (2019) ^[64]^ | 0.9NaNbO_3_ – 0.1Bi(Mg_2/3_Nb_1/3_)O_3_ | Bulk | 2.8 | 82 |
| J. Ye et al. (2019) ^[64]^ | 0.85NaNbO_3_ – 0.15Bi(Mg_2/3_Nb_1/3_)O_3_ | Bulk | 2.4 | 80 |
| W. Yang et al. (2022) ^[65]^ | Na_0.7_Bi_0.1_Nb_0.9_Ta_0.1_O_3_ | Bulk | 6.68 | 90.5 |
| J. Wang et al. (2021) ^[66]^ | 0.95NaNbO_3_ – 0.05La(Mn_0.5_Ni_0.5_)O_3_ | Bulk | 1.77 | 77 |
| J. Shi et al. (2020) ^[67]^ | 0.78NaNbO_3_ – 0.22Bi(Mg_2/3_Ta_1/3_)O_3_ | Bulk | 5.01 | 86.1 |
| M. Zhou et al. (2018) ^[68]^ | 0.8NaNbO_3_ – 0.2SrTiO_3_ | Bulk | 3.02 | 80.7 |
| J. Ma et al. (2022) ^[69]^ | 0.88NaNbO_3_ – 0.12(Bi_0.9_Na_0.1_)(Fe_0.8_Ti_0.2_)O_3_ | Bulk | 12.07 | 82.5 |
| R. Zhao et al. (2024) ^[70]^ | 0.85NaNbO_3_ – 0.15Bi(Mg_0.2_Hf_0.2_Ni_0.2_Zn_0.2_Ta_0.2_)O_3_ | Bulk | 5.11 | 77 |
| L.-F. Zhu et al. (2021) ^[71]^ | 0.8NaNbO_3_ – 0.04CaZrO_3_ – 0.16Bi_0.5_Na_0.5_TiO_3_ | Bulk | 3.7 | 82.1 |
| P. Zhao et al. (2022) ^[72]^ | 0.6BaTiO_3_ – 0.4NaNbO_3_ | Bulk | 3.07 | 92.6 |
| H. Dong et al. (2022) ^[25]^ | Na_0.9_NbO_3_ | Thin Film | 5.32 | 60 |
| Y. Zhang et al. (2024) ^[73]^ | NaNbO_3_ | Thin Film | 3.6 | 68.31 |
|  |  |  |  |  |
